# A Genome-Wide mRNA Screen and Functional Analysis Reveal *FOXO3* as a Candidate Gene for Chicken Growth

**DOI:** 10.1371/journal.pone.0137087

**Published:** 2015-09-14

**Authors:** Biao Chen, Jiguo Xu, Xiaomei He, Haiping Xu, Guihuan Li, Hongli Du, Qinghua Nie, Xiquan Zhang

**Affiliations:** 1 Department of Animal Genetics, Breeding and Reproduction, College of Animal Science, South China Agricultural University, Guangzhou, 510642, Guangdong, China; 2 Guangdong Provincial Key Lab of Agro-Animal Genomics and Molecular Breeding and Key Lab of Chicken Genetics, Breeding and Reproduction, Ministry of Agriculture, Guangzhou, 510642, Guangdong, China; 3 School of Bioscience and Bioengineering, South China University of Technology, Guangzhou, 510006, China; University of Bonn, GERMANY

## Abstract

Chicken growth performance provides direct economic benefits to the poultry industry. However, the underlying genetic mechanisms are unclear. The objective of this study was to identify candidate genes associated with chicken growth and investigate their potential mechanisms. We used RNA-Seq to study the breast muscle transcriptome in high and low tails of Recessive White Rock (WRR_h_, WRR_l_) and Xinghua chickens (XH_h_, XH_l_). A total of 60, 23, 153 and 359 differentially expressed genes were detected in WRR_h_ vs. WRR_l_, XH_h_ vs. XH_l_, WRR_h_ vs. XH_h_ and WRR_l_ vs. XH_l_, respectively. GO, KEGG pathway and gene network analyses showed that *CEBPB*, *FBXO32*, *FOXO3* and *MYOD1* played key roles in growth. The functions of *FBXO32* and *FOXO3* were validated. *FBXO32* was predominantly expressed in leg muscle, heart and breast muscle. After decreased *FBXO32* expression, growth-related genes such as *PDK4*, *IGF2R* and *IGF2BP3* were significantly down-regulated (P < 0.05). *FBXO32* was significantly (P < 0.05) associated with carcass and meat quality traits, but not growth traits. *FOXO3* was predominantly expressed in breast and leg muscle. In both of these tissues, the *FOXO3* mRNA level in XH was significantly higher than that in WRR chickens with normal body weight (P < 0.05). In DF-1 cells, siRNA knockdown of *FOXO3* significantly (P < 0.01) inhibited the *MYOD* expression and significantly up-regulated (P < 0.01 or P < 0.05) the expression of growth-related genes including *CEBPB*, *FBXO32*, *GH*, *GHR*, *IGF1R*, *IGF2R*, *IGF2BP1*, *IGF2BP3*, *INSR*, *PDK1* and *PDK4*. Moreover, 18 SNPs were identified in *FOXO3*. G66716193A was significantly (P < 0.05) associated with growth traits. The sites C66716002T, C66716195T and A66716179G were significantly (P < 0.05) associated with growth or carcass traits. These results demonstrated that *FOXO3* is a candidate gene influencing chicken growth. Our observations provide new clues to understand the molecular basis of chicken growth.

## Introduction

Chicken growth, an important economic trait, is determined by genetic, nutritional and environmental factors. Heritability estimates showed that chicken growth could be enhanced by genetic improvement [1, 2]. This trait is controlled by multiple genes. At present, many studies have been performed to find genetic factors affecting growth. Candidate genes and quantitative trait loci (QTLs) such as *GH*, *IGFBP2* and *GHSR* have been identified [3, 4]. Recently, genome-wide associate study (GWAS) analysis found that two *FOXO1A* single-nucleotide polymorphisms (SNPs) were strongly associated with chicken growth [5]. However, the genetic mechanisms of chicken growth are unclear, and a more systematic picture of the genes responsible for this trait is needed. Recently, next generation sequencing provided an important opportunity for the genome-wide characterization of genes and pathways involved in growth [6–8].

In this study, two chicken breeds, Recessive White Rock (WRR) and Xinhua (XH), were used for RNA-Seq. WRR is a typical fast-growing breed that is known for its large body size and thick myofibers. XH is a Chinese native slow-growing breed which is characterized by small body size. The different growth speeds of these two breeds led to distinct growth performance at 7 weeks of age. Both breeds were used for studies on chicken growth and fat deposition traits [1, 9]. With the use of a population derived from reciprocal crosses between these two breeds, a quantitative trait loci (QTL) on chromosome 1 was identified to be related to chicken growth traits by Genome-wide association study [5]. Previous study by Methylated DNA immunoprecipitation-sequencing showed that growth-related genes exhibited altered DNA methylation between WRR and XH [10]. Therefore, in the present study, we intended to use two-tail samples of these two breeds at 7 weeks of age to study the gene expression differences between fast- and slow-growing broilers in a genome-wide level and then to identify candidate genes for chicken growth. In our study, we performed RNA-Seq and differentially expressed genes (DEGs) were randomly selected to conduct qPCR experiments to validate the RNA-Seq results. Gene ontology (GO), Kyoto Encyclopedia of Genes and Genomes (KEGG) pathway and gene network analyses were performed on the DEGs. Subsequently, the chicken *FOXO3* and *FBXO32* genes were selected for *in vivo* and *in vitro* studies to investigate their potential mechanisms functioned on growth.

## Results

### Assemble and blast analysis of reads from RNA-Seq

**Fig 1:**
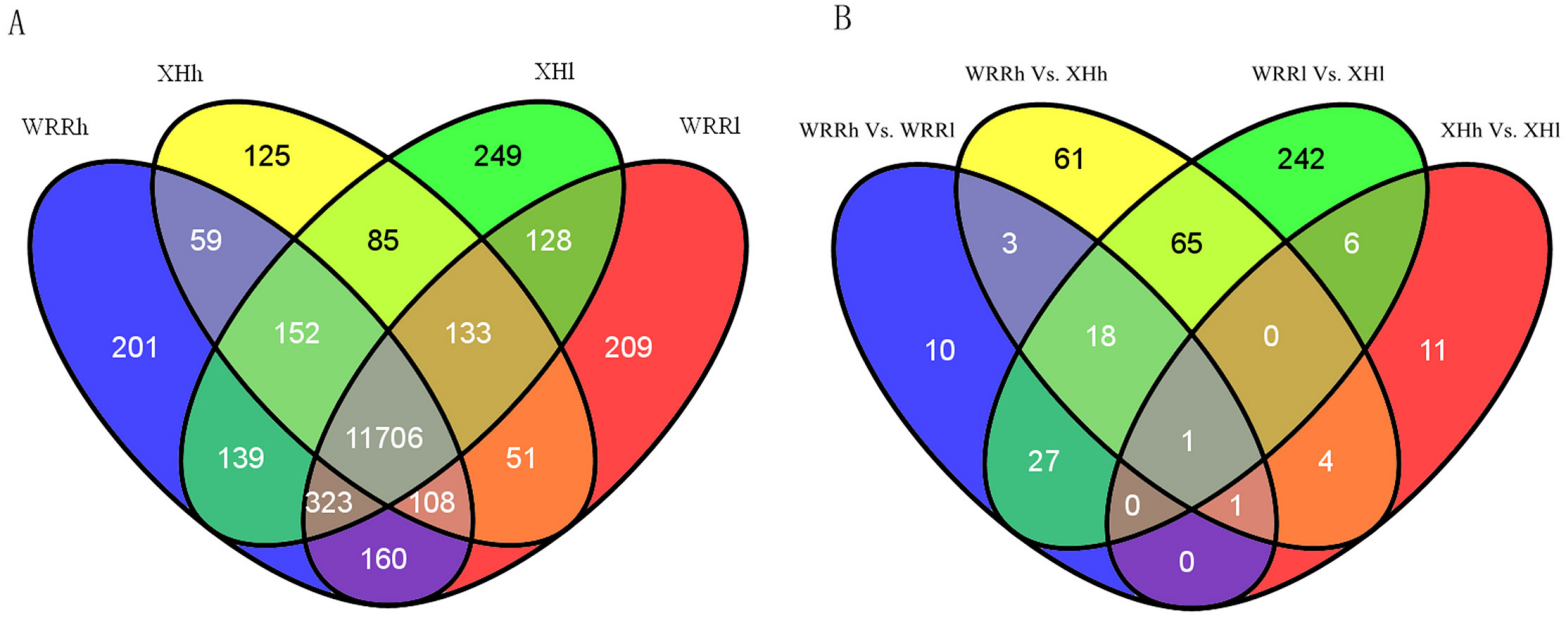
Numbers of expressed genes and differentially expressed genes: results of RNA-Seq. A: Expressed genes among four samples. B: Differentially expressed genes among four contrasts including WRR_h_ Vs. WRR_l_, XH_h_ Vs. XH_l_, WRR_h_ Vs. XH_h_ and WRR_l_ Vs. XH_l._ WRR_h_, WRR_l_, XH_h_ and XH_l_ indicated the group of Recessive White Rock with high body weight, Recessive White Rock with low body weight, Xinghua with high body weight and Xinghua with low body weight, respectively. The gene or differentially expressed gene number was shown at the top of each figure section.

From RNA-Seq, we obtained 44139971, 36937542, 39046772 and 69669990 Illumina reads for WRR_h_, WRR_l_, XH_h_ and XH_l_, respectively, giving rise to total residues of 4286130490, 3586054428, 3758078838 and 6698804887 bp, respectively (Table 1). All sequencing data have been submitted to NCBI Gene Expression Omnibus (GEO) database with the accession number GSE72424 (http://www.ncbi.nlm.nih.gov/geo/query/acc.cgi?acc=GSE72424). More than 70.5% of the total reads were mapped to the chicken genome. In total, 13828 genes were detected in the four samples, including 12848 in WRR_h_, 12818 in WRR_l_, 12419 in XH_h_ and 12915 in XH_l_ (Fig 1A). Of these genes, 11706 genes were identified in all four samples, while 201, 209, 125 and 249 genes were found exclusively in WRR_h_, WRR_l_, XH_h_ and XH_l_, respectively (Fig 1A). The sequence length distribution showed that 83% of genes identified in the four samples had a length less than 4000 bp, while no more than 0.5% of genes were longer than 10000 bp (S1 Fig).

**Table 1:** Data generated from RNA-seq.

Sample^1^	Total number of reads	Total residues (bp)	Total mapped reads	Percentage of mapped reads^2^
WRR_h_	44139971	4286130490	32685128	74.0%
WRR_l_	36937542	3586054428	27449227	74.3%
XH_h_	39046772	3758078838	27532015	70.5%
XH_l_	69669990	6698804887	49272817	70.7%

^1^WRR_h_, WRR_l_, XH_h_ and XH_l_ indicated the group of Recessive White Rock with high body weight, Recessive White Rock with low body weight, Xinhua Chickens with high body weight and Xinhua Chickens with low body weight, respectively.

^2^Percentage of mapped reads in total reads.

### DEGs among the four groups

A comparison of gene expression among the four samples showed that there were 60 DEGs (fold changes ≥ 2; q value < 0.05) between WRR_h_ and WRR_l_ (WRR_h_ vs. WRR_l_), 23 DEGs between XH_h_ and XH_l_ (XH_h_ vs. XH_l_), 153 DEGs between WRR_h_ and XH_h_ (WRR_h_ vs. XH_h_), and 359 DEGs between WRR_l_ and XH_l_ (WRR_l_ vs. XH_l_) (Fig 1B and S1 Table). *LAC_CHICK* (Ig lambda chain V-1 region) was found in all four comparisons. *LAC_CHICK* and *TTR* (transthyretin) were commonly identified in WRR_h_ vs. WRR_l_ and XH_h_ vs. XH_l_ (S2 Table). Moreover, 84 genes, including some crucial to chicken growth such as *FBXO32*, *FOXO3*, *MYOD1*, *PDK4*, *PNPLA2* and *SMYD1*, were commonly identified in WRR_h_ vs. XH_h_ and WRR_l_ vs. XH_l_ (S3 Table). Among all these DEGs, 6, 10, 11 and 12 genes were uniquely expressed in one of the two samples in each comparison of WRR_h_ vs. WRR_l_, XH_h_ vs. XH_l_, WRR_h_ vs. XH_h_ and WRR_l_ vs. XH_l_, respectively (S4 Table). DEG directionality analysis showed that the number of up-regulated genes was higher than the number of down-regulated genes in both WRR_h_ vs. WRR_l_ and XH_h_ vs. XH_l_, while there were a greater number of down-regulated genes than of up-regulated genes in both WRR_h_ vs. XH_h_ and WRR_l_ vs. XH_l_ (S2 Fig).

### qPCR validation of DEGs obtained from RNA-Seq

To confirm the DEG results obtained from RNA-Seq, four genes (*RPL29*, *PDK4*, *FOXO3* and *LAPTM5*) were randomly selected to carry out qPCR using the same RNA samples used for RNA-Seq. The qPCR results showed general agreement with the RNA-Seq results in terms of the direction of expression in each comparison (Table 2).

**Table 2:** qPCR validation of DEGs obtained from RNA-seq.

Gene	Contrast	qPCR^1^	RNA-seq^2^
RPL29	WRR_h_ Vs. WRR_l_	1.58	1.05
	XH_h_ Vs. XH_l_	1.09	1.04
	WRR_h_ Vs. XH_h_	1.42	0.90
	WRR_l_ Vs. XH_l_	0.98	0.89
PDK4	WRR_h_ Vs. WRR_l_	7.26	4.60
	XH_h_ Vs. XH_l_	3.03	1.94
	WRR_h_ Vs. XH_h_	4.89	13.96
	WRR_l_ Vs. XH_l_	2.04	5.90
FOXO3	WRR_h_ Vs. WRR_l_	7.82	2.18
	XH_h_ Vs. XH_l_	0.76	0.98
	WRR_h_ Vs. XH_h_	11.13	6.56
	WRR_l_ Vs. XH_l_	1.08	2.96
LAPTM5	WRR_h_ Vs. WRR_l_	11.47	2.18
	XH_h_ Vs. XH_l_	2.18	1.39
	WRR_h_ Vs. XH_h_	4.63	0.32
	WRR_l_ Vs. XH_l_	0.88	0.20

^1^meant the ratio of expression value (2^-ΔΔCt^) in the second group to the first group,

^2^fold change between contrasts presented (the ratio of FPKM in the second group to the first group).

### GO and KEGG pathway analysis for DEGs

DEGs were then used for GO analysis to uncover enriched (P < 0.05) biological processes terms in each comparison. A total of 142 biological process terms, including 21 in WRR_h_ vs. WRR_l_, 6 in XH_h_ vs. XH_l_, 47 in WRR_h_ vs. XH_h_ and 119 in WRR_l_ vs. XH_l_, were identified in our study (S5 Table). Of these, 99 were unique terms that appeared only once in all of the four comparisons. Many of the repeated biological process terms were focused on developmental process, regulation of biological process, cell differentiation and cell adhesion. A total of 29 DEGs involved in cell differentiation and proliferation were observed in the four comparisons, including well-known genes affecting chicken growth such as *CEBPB*, *MYH11*, *MYOD1*, *NOTCH2* and *TGFBR2* (Table 3). Moreover, in comparing XH_h_ vs. XH_l_ and WRR_h_ vs. XH_h_, the following processes related to muscle development were found: skeletal muscle development, muscle organ development, muscle cell differentiation and muscle tissue development (S5 Table). Six DEGs were included in those processes: *ACTC1*, *FOXP2*, *LGALS1*, *MYOD1*, *XIRP1* and *ZFAND5*. These genes might be crucial to muscle development. DEGs we identified were significant enriched (P < 0.05 and Benjiamini adjusted P < 0.1) in eight KEGG pathways, with the most influenced pathway being lysosome (Table 4). Furthermore, cell junction-related pathways such as focal adhesion, extracellular matrix (ECM)-receptor interaction and cytokine-cytokine receptor interaction were included. There were 45 DEGs in these four pathways, including *TGFBR2* and *ITGAV* (Table 5).

**Table 3:** Differentially expressed genes involved in the processes of cell differentiation and proliferation in the four comparisons.

Gene name	Description	Fold change (P value)
Actc1	actin,alpha,cardiac muscle 1; actin,alpha 1,skeletal muscle	4.6673034 (1.20E-06)
ANG	leukocyte ribonuclease A-1; leukocyte ribonuclease A-2	3.5635050(7.02E-09)
ANXA1	annexin A1	5.0952624(8.32E-09)
B-MA1	B locus M alpha chain 1	3.5732686(0.000159)
btk	Bruton agammaglobulinemia tyrosine kinase	3.5233038(0.002987)
CD3E	CD3e molecule, epsilon (CD3-TCR complex)	5.5777273(5.02E-08)
CD74	CD74 molecule,major histocompatibility complex	2.2219375(0.000123)
CEBPB	CCAAT/enhancer binding protein (C/EBP), beta	3.5283824(1.13E-05)
CTGF	connective tissue growth factor	4.2948028(2.72E-06)
CXCR4	chemokine (C-X-C motif) receptor 4	3.9855519(0.000613)
Dpysl2	dihydropyrimidinase-like 2	2.2867636(0.000232)
EFHD1	EF-hand domain family, member D1	3.2853895(3.68E-05)
FABP4	fatty acid binding protein 4, adipocyte	5.4548149(3.38E-10)
Fkbp1b	FK506 binding protein 1B, 12.6 kDa	2.9395742(0.000495)
FYN	FYN oncogene related to SRC, FGR, YES	2.9063163(1.53E-05)
LGALS1	lectin, galactoside-binding, soluble, 1	8.8038404(1.47E-12)
LIPA	lipase A lysosomal acid cholesterol esterase	9.7054782(4.66E-15)
LYN	v-yes-1 Yamaguchi sarcoma viral related oncogene homolog	4.8534220(3.98E-08)
MDK	midkine (neurite growth-promoting factor 2)	8.3954349(1.06E-07)
MYH11	myosin heavy chain 10 non-muscle; myosin heavy chain 11	2.3873684(9.77E-07)
MYOD1	myogenic differentiation 1	8.3954349(1.06E-07)
Notch2	Notch homolog 2 (Drosophila)	2.1351327(0.000257)
plek	pleckstrin	5.8407637(1.37E-07)
Ptprc	protein tyrosine phosphatase receptor type C	4.1222595(9.99E-14)
sfrp2	secreted frizzled-related protein 2	6.4703796(4.28E-08)
Tgfbr2	transforming growth factor, beta receptor II (70/80kDa)	2.1053138(0.000330)
THY1	Thy-1 cell surface antigen	7.3370184(4.45E-12)
TXNRD1	thioredoxin reductase 1	3.1127232(0.000165)
XIRP1	xin actin-binding repeat containing 2	4.0403702(5.75E-08)

**Table 4:** Enriched KEGG pathways for all DEGs identified in the four comparisons.

No.	Pathways	P value	Benjiamini
1	Lysosome	1.2E-07	9.4E-06
2	Focal adhesion	0.0041	0.15
3	Drug metabolism	0.0045	0.12
4	Metabolism of xenobiotics by cytochrome P450	0.024	0.39
5	ECM-receptor interaction	0.027	0.36
6	Cytokine-cytokine receptor interaction	0.03	0.33
7	Intestinal immune network for IgA production	0.039	0.37
8	Glutathione metabolism	0.048	0.39

P < 0.05 and Benjiamini adjusted p < 0.1 was regarded as enriched.

**Table 5:** Differentially expressed genes involved in the five pathways related to growth.

Gene	Description	Gene	Description
actn1	actinin, alpha 1	fn1	fibronectin 1
Atp6v0d2	ATPase, H+ transporting, lysosomal 38kDa, V0 subunit D2	FYN	FYN oncogene related to SRC, FGR, YES
CCR2	chemokine (C-C motif) receptor 2	GNPTAB	N-acetylglucosamine-1-phosphate transferase,alpha and beta subunits
CCR5	chemokine (C-C motif) receptor 5	GUSB	glucuronidase, beta
CD44	CD44 molecule	il2rg	interleukin 2 receptor, gamma
CHAD	chondroadherin	Itgav	integrin, alpha V
CLTA	clathrin, light chain (Lca)	LAMP3	lysosomal-associated membrane protein 3
Col5a1	collagen, type V, alpha 1	LAPTM4A	lysosomal-associated protein transmembrane 4 alpha
Col6a3	collagen, type VI, alpha 3	LAPTM5	lysosomal associated multispanning membrane protein 5
comP	cartilage oligomeric matrix protein	LGMN	legumain
CSF2RA	colony stimulating factor 2 receptor, alpha	lipA	lipase A, lysosomal acid, cholesterol esterase
csf3r	colony stimulating factor 3 receptor	LYPLA3	lysophospholipase 3
CTSA	cathepsin A	PARVB	parvin, beta
CTSB	cathepsin B	pdgfra	platelet-derived growth factor receptor, alpha polypeptide
CTSC	cathepsin C	Pdgfrb	platelet-derived growth factor receptor, beta polypeptide
CTSH	cathepsin H	Pik3cb	phosphoinositide-3-kinase, catalytic, beta polypeptide
CTSL2	cathepsin L2	Samd13	deoxyribonuclease II beta;sterile alpha motif domain containing 13
CTSS	cathepsin S	SPP1	secreted phosphoprotein 1
CXCL12	chemokine (C-X-C motif) ligand 12	TCIRG1	T-cell, immune regulator 1, ATPase, H+ transporting
CXCL13L2	similar to macrophage inflammatory protein-2	Tgfbr2	transforming growth factor, beta receptor II (70/80kDa)
CXCL14	chemokine (C-X-C motif) ligand 14	TLN1	talin 1
CXCR4	chemokine (C-X-C motif) receptor 4	ZYX	zyxin
FLNB	filamin B, beta		

### Gene network analysis for DEGs

**Fig 2:**
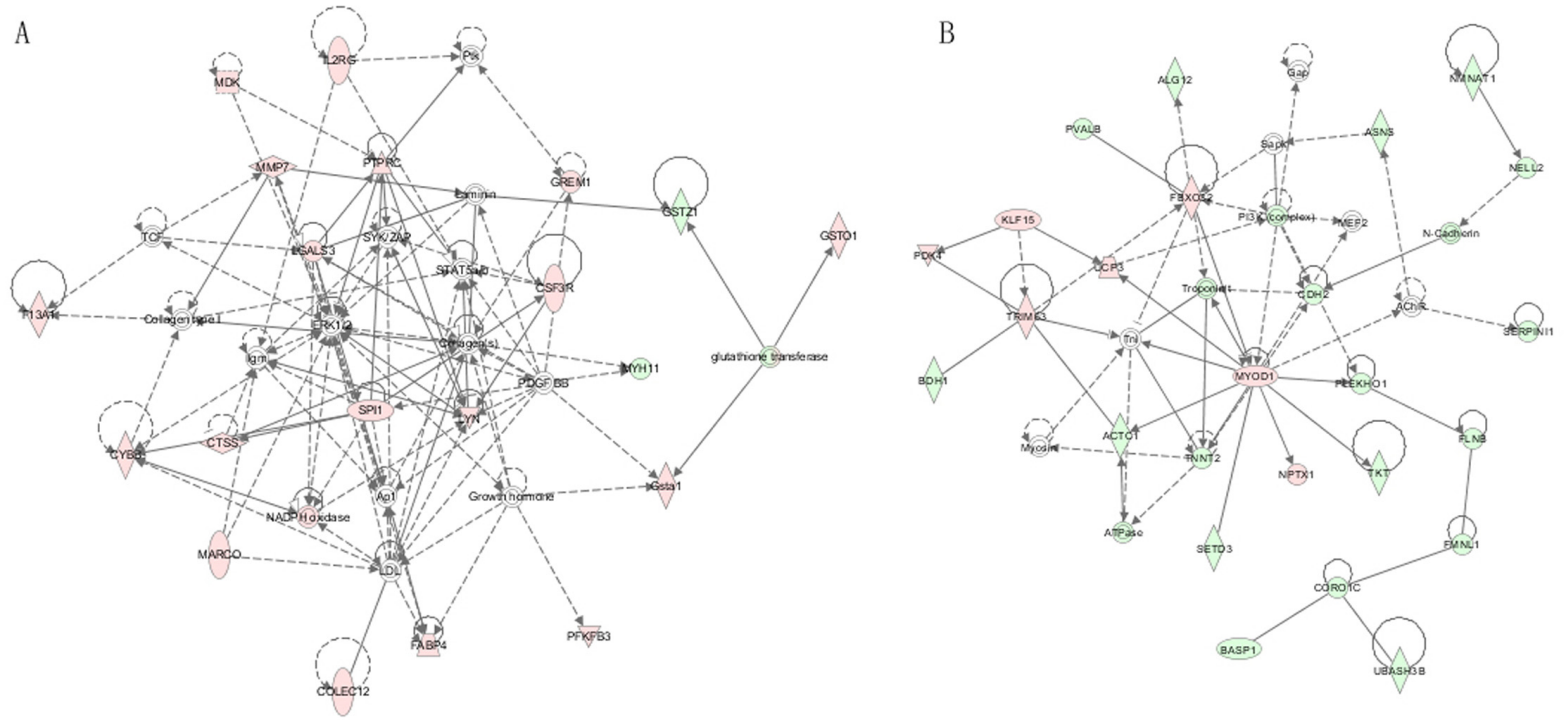
The top gene network in the inner contrasts and across-breed contrasts. A: The top gene network identified in inner contrasts (WRR_h_ Vs. WRR_l_ and XH_h_ Vs. XH_l_). B: The top gene network identified in across-breed contrasts (WRR_h_ Vs. XH_h_ and WRR_l_ Vs. XH_l_). Genes exhibiting up-regulated were shown in red, while gene exhibiting down-regulated were shown in green color. The color intensity indicated the degree of up-/down-regulated. Solid lines and dashed lines indicated direct interaction and indirect interaction, respectively.

Subsequently, we used Ingenuity Pathway Analysis (IPA, Ingenuity Systems; http://www.ingenuity.com) to investigate the gene networks for DEGs identified in the inner comparisons (WRR_h_ vs. WRR_l_ and XH_h_ vs. XH_l_). The results showed that the top gene network was cellular movement (Fig 2A). Among the DEGs, *FABP4*, *LGALS3*, *LYN* and *SPI1* were central of this network. Network analysis for all DEGs in the cross-breed comparisons (WRR_h_ vs. XH_h_ and WRR_l_ vs. XH_l_) revealed that the top gene network was skeletal and muscular system development and function (Fig 2B), with *MYOD1* and *FBXO32* as node genes.

**Fig 3:**
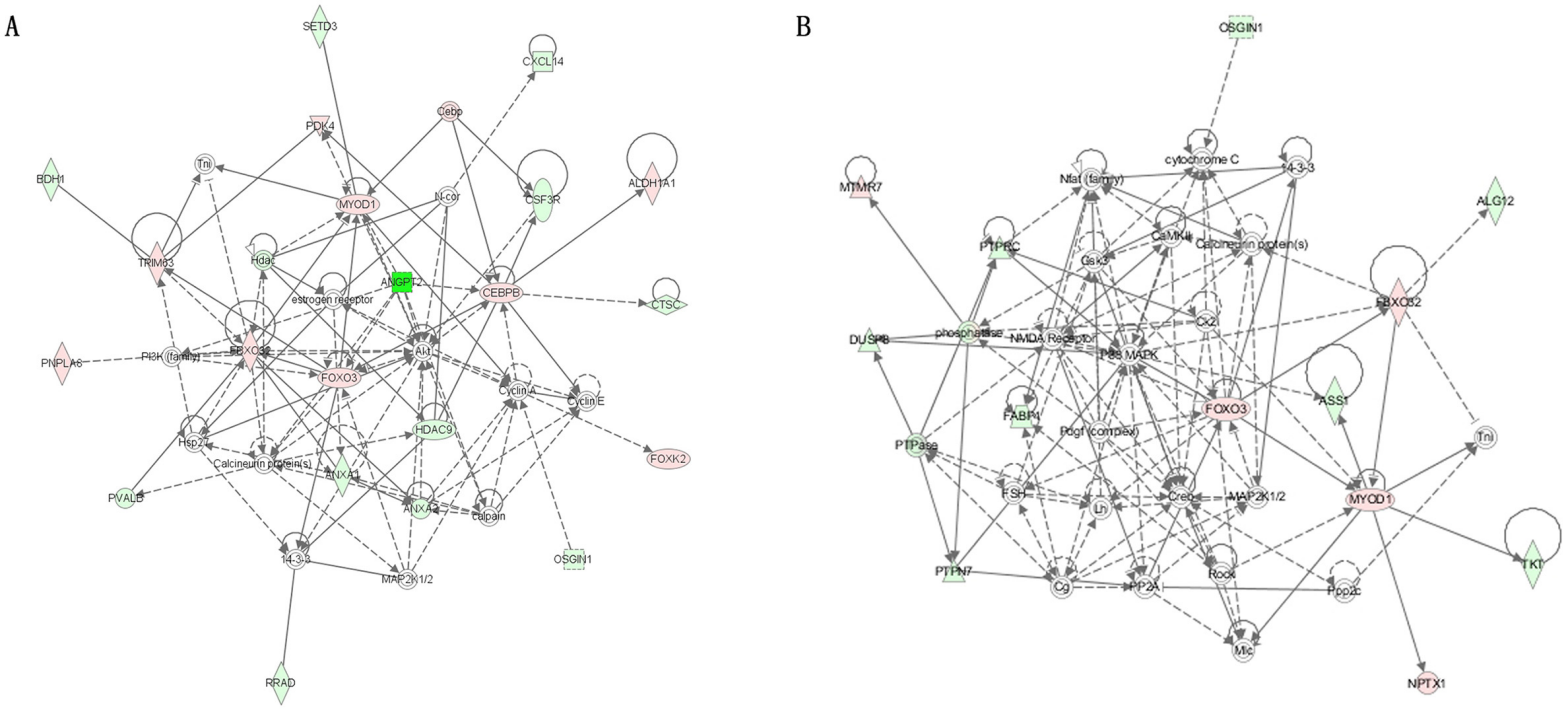
Gene networks involved the *FBXO32* and *MYOD1* in WRR_h_ Vs. XH_h_ and WRR_l_ Vs. XH_l_. A: in WRR_h_ Vs. XH_h_. B: in WRR_l_ Vs. XH_l_. Genes exhibiting up-regulated were shown in red, while gene exhibiting down-regulated were shown in green color. The color intensity indicated the degree of up-/down-regulated. Solid lines and dashed lines indicated direct interaction and indirect interaction, respectively.

The results of the GO, KEGG pathway and gene network analyses indicated that *CEBPB*, *FBXO32* and *MYOD1* might be the key genes related to chicken growth at 7 weeks of age. Further investigation of gene networks involving these three genes showed that *FOXO3* might be the crucial gene, interacting with *CEBPB*, *FBXO32*, and *MYOD1* and to affect growth (Fig 3A and 3B, which show networks compared for WRR_h_ vs. XH_h_ and WRR_l_ vs. XH_l_, respectively). In WRR_h_ vs. XH_h_ and WRR_l_ vs. XH_l_, the *CEBPB*, *FBXO32*, *FOXO3* and *MYOD1* mRNA levels were all higher in slow-growing chickens than in fast-growing chickens (Table 6). Furthermore, *FBXO32* and *FOXO3* showed relatively high fold changes in both of these two comparisons. Therefore, *FBXO32* and *FOXO3* were selected for functional analysis in this study.

**Table 6:** Differential expression of *CEBPB*, *FBXO32*, *FOXO3* and *MYOD1* in contrasts of WRR_h_ Vs. XH_h_ and WRR_l_ Vs. XH_l._

Gene	WRR_h_ FPKM	XH_h_ FPKM	log_2_Ratio (XH_h_/ WRR_h_)	WRR_l_ FPKM	XH_l_ FPKM	log_2_Ratio (XH_l_/ WRR_l_)
*CEBPB*	45.09	159.10	1.82	62.82	138.74	1.14
*FBXO32*	23.94	208.70	3.12	21.52	235.93	3.45
*FOXO3*	14.13	92.67	2.71	30.76	90.89	1.56
*MYOD1*	118.89	277.56	1.22	112.65	313.63	1.48

### cDNA clone of the chicken *FOXO3* gene

Using PCR amplification and 3’ rapid amplification of cDNA ends (3’RACE), a 2,882 bp (or 1,874 bp) cDNA fragment of the chicken *FOXO3* gene, including the 1,443 bp CDS and the full-length 3’ UTR (1,439 bp or 431 bp), was obtained (S3 Fig). The chicken FOXO3 showed more than 85.4% identity with its human, mouse, rat, elephant, whale, pig, dog, sheep and cattle, counterparts. However, much lower homology with cFOXO3 was found for fish and xenopus, which were 47.4% and 77.1%, respectively.

### *FOXO3* and *FBXO32* gene mRNA expression in different tissues and between different breeds

**Fig 4:**
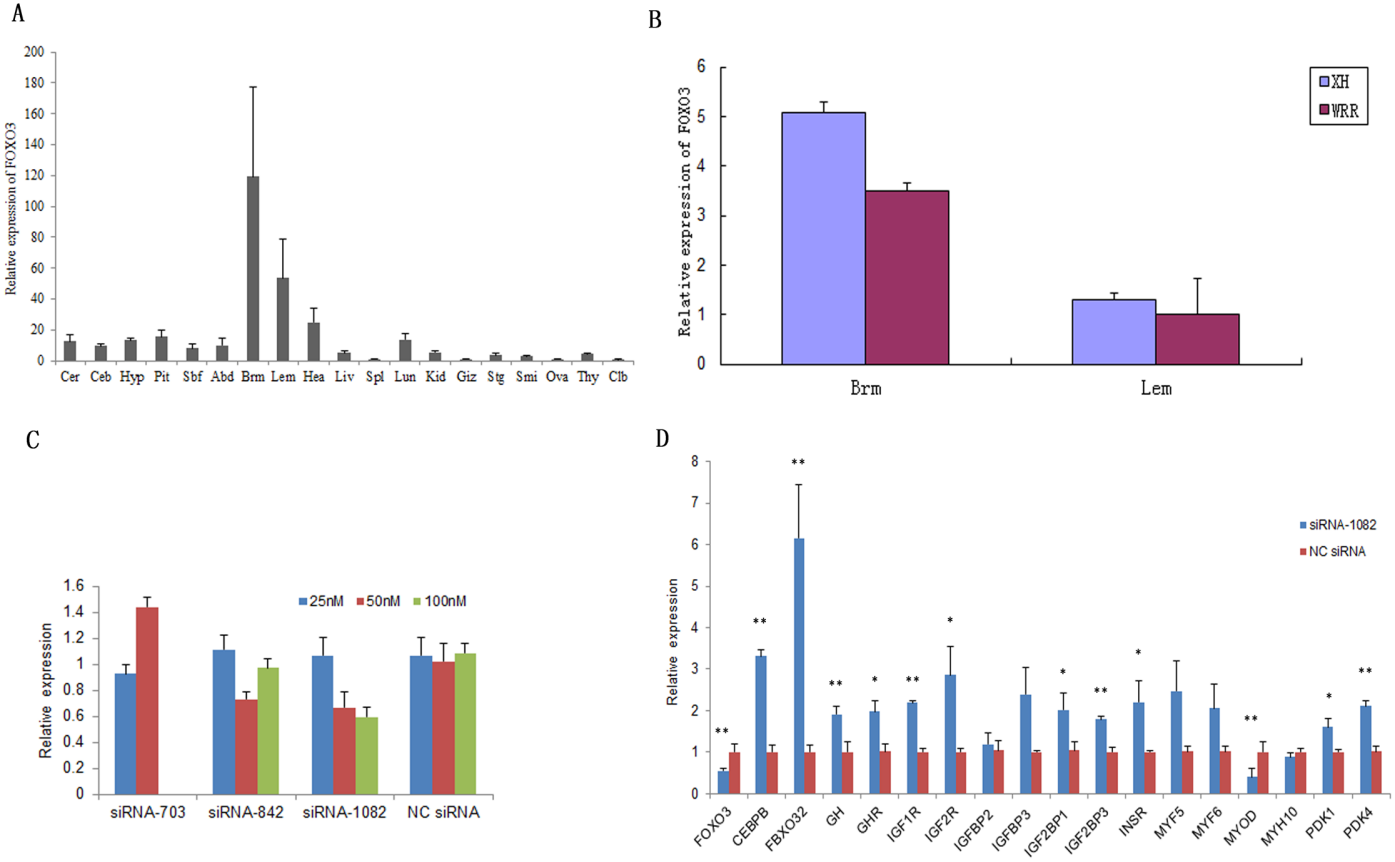
*FOXO3* expression in different tissues, different breeds, DF-1 cells and the up/down-regulation of growth-related genes. A: The mRNA level of *FOXO3* in different tissues. Cer: cerebrum, Ceb: cerebellum, Hyp: hypothalamus, Pit: pituitary, Sbf: subcutaneous fat, Abd: abdominal fat, Brm: breast muscle, Lem: leg muscle, Hea: heart, Liv: liver, Spl: spleen, Lun: lung, Kid: kidney, Giz: gizzard, Stg: stomachus glandularis, Smi: small intestine, Ova: vary, Thy: thymus, Clb: cloacal bursa. The horizontal axis indicated different tissues, while the vertical axis indicated 2^-ΔΔCt^ value. B: The expression difference of *FOXO3* between XH and WRR. C: Relative expression of *FOXO3* in DF-1 cells transfected with different siRNAs. The x-axis described siRNAs and the y-axis showed the relative expression of *FOXO3*. D: The relative expression changes of growth related genes in DF-1 transfected with siRNA-1082 at 100 nM. **and*indicated P < 0.01 and P < 0.05, respectively.

**Fig 5:**
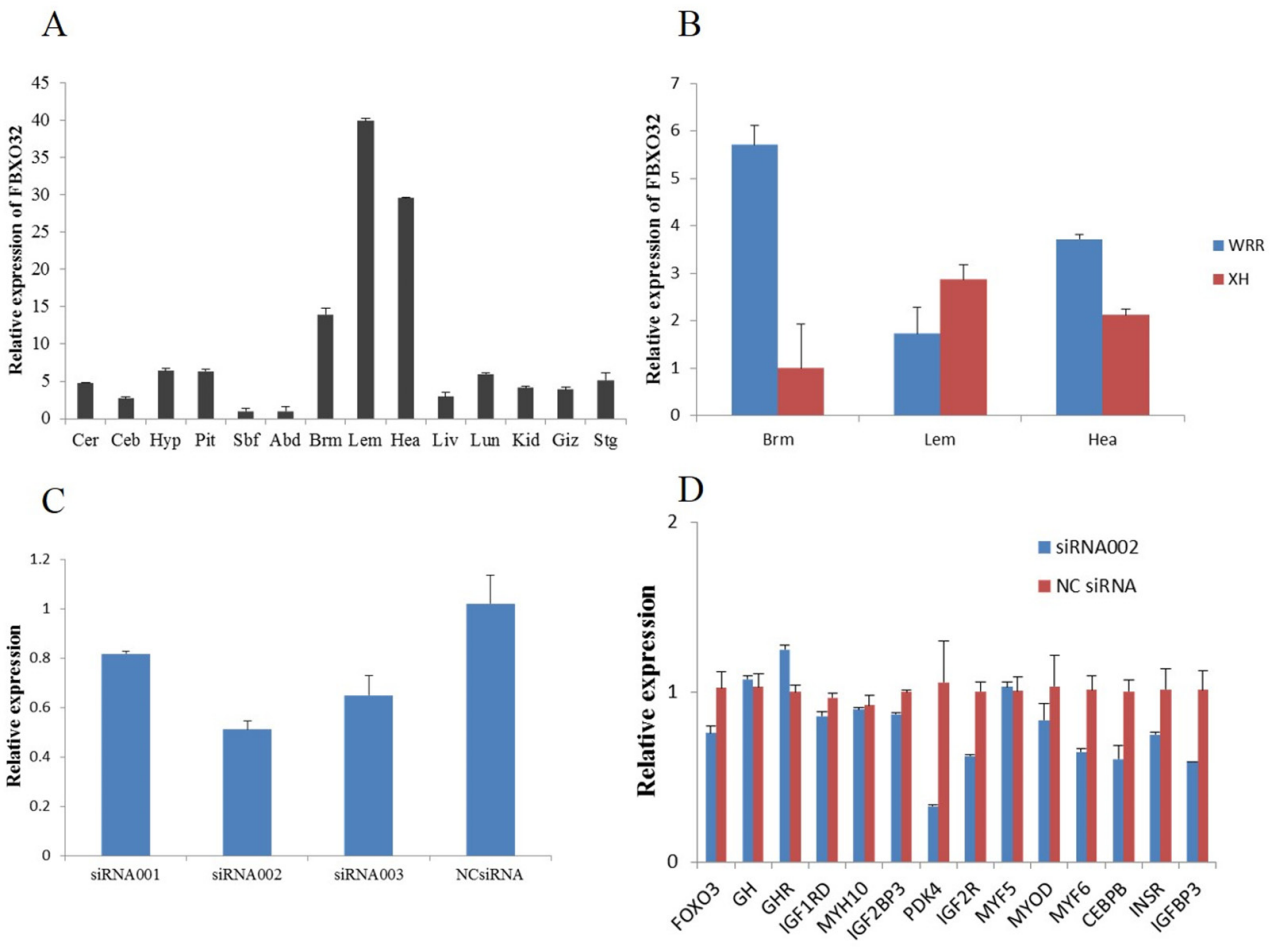
*FBXO32* expression in different tissues, different breeds, DF-1 cells and the up/down-regulation of growth-related genes. A: The mRNA level of *FBXO32* in different tissues in XH chickens. Cer: cerebrum, Ceb: cerebellum, Hyp: hypothalamus, Pit: pituitary, Sbf: subcutaneous fat, Abd: abdominal fat, Brm: breast muscle, Lem: leg muscle, Hea: heart, Liv: liver, Lun: lung, Kid: kidney, Giz: gizzard, Stg: stomachus glandularis. The horizontal axis indicated different tissues, while the vertical axis indicated 2^-ΔΔCt^ value. B: The expression difference of *FBXO32* between XH and WRR. C: Relative expression of *FBXO32* in DF-1 cells transfected with different siRNAs. The x-axis described siRNAs and the y-axis showed the relative expression of *FBXO32*. D: The relative expression changes of growth related genes in DF-1 transfected with siRNA002 at 50 nM. **and*indicated P < 0.01 and P < 0.05, respectively.

The mRNA expression analysis in various tissues showed that the chicken *FOXO3* gene was predominantly expressed in breast muscle and leg muscle tissues, followed by heart, lung and pituitary (Fig 4A). *FOXO3* expression was compared between WRR and XH chickens with normal body weight (BW) at seven weeks of age. In breast muscle, its mRNA level was significantly higher in XH chickens (P < 0.01) than in WRR chickens (Fig 4B). In leg muscle, expression in XH was significantly higher (P < 0.05) than in WRR (Fig 4B). The chicken *FBXO32* gene was predominantly expressed in leg, heart and breast muscle (Fig 5A). The mRNA level of *FBXO32* was significantly higher in breast and heart muscle than in XH (P < 0.05) but lower in leg muscle (P < 0.05) (Fig 5B).

### The expression change of growth-related genes after RNA interference of candidate genes

To find suitable conditions for subsequent RNA interference experiments, we first evaluated the cell activity after transfection. From 5 to 48 h post-transfection, cell activity was not reduced and apoptosis did not occur in either DF-1 cells (S4A Fig) and skeletal muscle cell (S4B Fig). Moreover, the cell density became stronger with the advancement of time. Therefore, the transfected cells were collected at 48 h post transfection for further analysis.

To investigate the interfering efficiency of different small interfering RNAs (siRNAs), three specific siRNAs targeting *FOXO3*, siRNA-703, siRNA-842 and siRNA-1082 were transfected into DF-1 cells at three different concentrations (25 nM, 50 nM and 100 nM). Cells treated with non-specific siRNA (NC siRNA) were used as a control. The transfection of DF-1 cells with 25 nM siRNA-703 led to a 10% decrease in *FOXO3* gene expression, whereas transfection with 25 nM siRNA-842 or siRNA-1082 had no effect on gene expression (Fig 4C). After transfection with 50 nM siRNA-703, gene expression was not decreased. After transfection of siRNA-842 or siRNA-1082 at 50 nM, gene expression decreased 30% (Fig 4C). The best knockdown was mediated by siRNA-1082 transfection at 100 nM, resulting in 41% reduced gene expression (Fig 4C). For *FBXO32*, siRNA-001, siRNA-002 and siRNA-003 were synthesized and transfected into DF-1 cells at 50 nM which is the recommended concentration for Lipofectamine 2000. NC siRNA was used as a control at 50 nM. The three siRNAs decreased *FOXO3* gene expression by 18%, 49% and 35%, respectively (Fig 5C). Therefore, siRNA-002 was used for transfecting DF-1 cells.

Then, 100 nM siRNA-1082 was transfected into DF-1 cells. At 48 h after transfection, qPCR results showed that it effectively (P < 0.01) inhibited the expression of *FOXO3* (Fig 4D). *FOXO3* expression knockdown resulted in the significant up-regulation of many growth related genes, including *CEBPB* (P < 0.01), *FBXO32* (P < 0.01), *GH* (P < 0.01), *GHR* (P < 0.05), *IGF1R* (P < 0.01), *IGF2R* (P < 0.05), *IGF2BP1* (P < 0.05), *IGF2BP3* (P < 0.01), *INSR* (P < 0.05), *PDK1* (P < 0.05) and *PDK4* (P < 0.01), while the expression of *MYOD* was highly significantly down-regulated (P < 0.01). The mRNA levels of *IGFBP2*, *IGFBP3*, *MYF5*, *MYF6* and *MYH10* were not significantly changed (P > 0.05) (Fig 4D). The *IGF1* and *IGF2* expression levels were too low to be detected in DF-1 cells. After *FBXO32* gene expression was significantly decreased, many growth-related genes, including *PDK4*, *IGF2R* and *IGFBP3*, were significantly down-regulated (P < 0.05). The *GH* and *GHR* expression levels tended to increase. (Fig 5D)

### Association of SNPs in both of *FOXO3* and *FBXO32* with chicken growth traits

A total of 18 SNPs were identified in the chicken *FOXO3* gene (Table 7). Four SNPs were located in the intron region and 11 were located in the coding region. Among these SNPs in the coding region, only one (G66715314C) was a non-synonymous variation (Ala → Pro). Then, 13 SNPs, C66716195T (rs314403820), G66716193A (rs317912153), A66716179G (rs15379322), A66716172G (rs15379320), T66716119C (rs15379317), A66716041G (rs15379315), C66716002T, G66715897A, C66715807T, C66715738T (rs317670452), G66715672A (rs317919122), A66715543G (rs15379310) and T66715378C (rs15379307), were selected for marker-trait association analyses (Table 8). The minor allele frequency for each SNP was greater than 0.05. Association analysis showed that G66716193A (rs317912153) was significantly (P < 0.05) (P < 0.01) associated with growth traits, including BW at different weeks, shank length at 70 days (SL70), shank length at 84 days (SL84), shank diameter at 70 days (SD70) and the average daily gain (ADG) from 4 to 8 weeks. Birds with the AG genotype had higher BW at various time points than those with the GG genotype. The site C66716002T was significantly (P < 0.05) associated with growth traits including BW at 14 days, 21 days, 77 days and SD70. Conversely, the polymorphic site C66716195T (rs314403820) was significantly (P < 0.05) associated with carcass traits, such as chest width (CW), dressed weight (DW), semi-eviscerated weight (SEW), eviscerated weight (EW) and breast muscle weight (BMW). The site A66716179G (rs15379322) had significant (P <0.05) association with SD70 and abdominal fat pad weight (AFW).

**Table 7:** Summary of SNPs identified in the chicken *FOXO3* gene.

NO.	SNP^1^	RefSNP	Region	Nucleotide change	AA change^2^
1	G66786154A	rs315777420	unknown	/	/
2	66785514GTTC Indel	/	unknown	/	/
3	G66785391A	/	unknown	/	/
4	C66716195T	rs314403820	Intron	/	/
5	G66716193A	rs317912153	Intron	/	/
6	A66716179G	rs15379322	Intron	/	/
7	A66716172G	rs15379320	Intron	/	/
8	T66716119C	rs15379317	Exon	CAT → CAC	H → H
9	A66716041G	rs15379315	Exon	CCA → CCG	P → P
10	C66716002T	/	Exon	GCC → GCT	A → A
11	G66715897A	/	Exon	TCG → TCA	S → S
12	C66715807T	/	Exon	AAC → AAT	N → N
13	C66715738T	rs317670452	Exon	GAC → GAT	D → D
14	G66715672A	rs317919122	Exon	CCG → CCA	P → P
15	A66715543G	rs15379310	Exon	ACA → ACG	T → T
16	T66715378C	rs15379307	Exon	TTT → TTC	F → F
17	G66715314C	/	Exon	GCC → CCC	A → P
18	C66715165T	rs317127440	Exon	AAC → AAT	N → N

^1^The number indicated the position on the chromosome 3.

^2^AA indicated amino acid.

**Table 8:** Association of SNPs in the chicken *FOXO3* gene with the growth and carcass traits.

SNP	Traits	P value	Least-square Means ± standard errors (SE)		
G66716193A	BW7 (g)	<1×10^−4^	56.86±1.02^A^(AA/52)	61.33±0.68^B^(AG/116)	58.18±0.60^A^(GG/220)
	BW14 (g)	0.0245	120.90±2.17^a^(AA/55)	126.00±1.40^b^(AG/124)	121.67±1.25^a^(GG/244)
	BW21 (g)	0.0326	206.49±4.23^ab^(AA/51)	214.55±2.64^a^(AG/125)	205.90±2.38^b^(GG/239)
	BW35 (g)	0.0455	433.45±9.91^ab^(AA/54)	446.95±6.38^a^(AG/122)	425.48±5.73^b^(GG/236)
	BW42 (g)	0.0316	561.83±13.13^ab^(AA/55)	585.67±8.54^a^(AG/122)	556.68±7.66^b^(GG/240)
	BW49 (g)	0.0408	691.84±15.63^ab^(AA/55)	721.30±10.11^a^(AG/124)	689.33±9.02^b^(GG/243)
	BW56 (g)	0.0104	843.66±18.33^b^(AA/55)	883.89±11.85^Aa^(AG/123)	838.64±10.54^Bb^(GG/243)
	BW70 (g)	0.0005	1124.73±27.26^AB^(AA/47)	1169.77±17.74^A^(AG/94)	1075.11±15.47^B^(GG/196)
	BW77 (g)	0.0127	1311.23±31.19^AB^(AA/48)	1360.08±20.28^A^(AG/95)	1279.17±17.67^B^(GG/197)
	BW84 (g)	0.0373	1461.47±40.97^ab^(AA/36)	1514.82±25.69^a^(AG/76)	1425.07±22.82^b^(GG/161)
	SL70 (mm)	0.0065	81.62±0.77^AB^(AA/47)	83.16±0.50^A^(AG/94)	81.09±0.43^B^(GG/196)
	SD70 (mm)	0.0415	9.50±0.13^ab^(AA/47)	9.55±0.08^a^(AG/94)	9.23±0.07^b^(GG/196)
	SL84 (mm)	0.0403	87.76±0.94^ab^(AA/36)	89.52±0.59^a^(AG/76)	87.66±0.52^b^(GG/161)
	ADG2 (g/day)	0.0144	19.15±0.53^b^(AA/55)	20.26±0.35^Aa^(AG/120)	18.99±0.30^Bb^(GG/240)
C66716002T	BW14 (g)	0.0326	122.52±0.93^a^(CC/332)	124.75±1.85^a^(TC/83)	136.29±5046^b^(TT/7)
	BW21 (g)	0.0361	208.33±1.76^a^(CC/325)	210.71±3.47^a^(TC/82)	234.99±10.25^b^(TT/7)
	BW77 (g)	0.0454	1302.25±13.26^a^(CC/267)	1341.32±26.25^ab^(TC/66)	1473.21±76.22^b^(TT/6)
	SD70 (mm)	0.0462	9.36±0.054 ^a^(CC/265)	9.58±0.11^ab^(TC/65)	9.92±0.30^b^(TT/6)
C66716195T	CW (mm)	0.0131	68.20±4.49^a^(CC/76)	62.43±5.199^b^(TC/163)	62.26±6.55^ab^(TT/183)
	DW (g)	0.0131	1347.10±157.76^a^(CC/76)	1136.51±182.62^b^(TC/163)	1119.53±230.26^ab^(TT/183)
	SEW (g)	0.0212	1227.26±142.5^a^9(CC/76)	1046.46±165.07^b^(TC/163)	1030.24±208.12^ab^(TT/182)
	EW (g)	0.0199	1097.35±125.03^a^(CC/76)	935.36±144.74^b^(TC/163)	918.04±182.49^ab^(TT/182)
	BMW (g)	0.0132	91.07±11.39^a^(CC/76)	79.69±13.12^b^(TC/163)	83.25±16.63^ab^(TT/183)
A66716179G	SD70(mm)	0.0286	9.49±0.07^A^(AA/204)	9.37±0.07^a^(AG/111)	9.00±0.17^Bb^(GG/22)
	AFW (g)	0.0478	24.94±9.62^a^(AA/251)	7.97±12.41^b^(AG/143)	1.87±19.63^ab^ (GG/227)

BW7, BW14, BW21, BW35, BW42, BW49, BW56, BW70, BW77 and BW84 indicated body weight at 7, 14, 21, 35, 42, 49, 56, 70 and 77 days, respectively. SL70 or SL84 = shank length at 70 or 84 days. SD70 = shank diameter at 70 days. ADG2 meant average daily gain from 4 to 8 weeks. CW = chest width. DW = dressed weight. SEW = semi-eviscerated weight. EW = eviscerated weight. BMW = breast muscle weight. AFW = abdominal fat pad weight. Number in brackets meant the number of birds tested for each genotype. The ^a,b^ or ^A,B^ values with no common superscripts within a column indicated the comparison was differed significantly (P < 0.05 or P < 0.01).

SNPs of chicken *FBXO32* were obtained from the NCBI SNP database. The 5’flank region is considered the promoter region of many genes. A total of 14 SNPs in the 5’flank region of *FBXO32* were chosen and confirmed by sequencing (Table 9). All of these SNPs, including A137669051T (rs314992955), C137669076T (rs313186471), C137669094T (rs741279417), C137669311G (rs733740424), C137669446T (rs16155707) A137669484C (rs737463671), A137669551G (rs316423136), A137669618G (rs731278593), C137669630T (rs315617235), A137669638T (rs313162537), C137669668G (rs317677782), A137669706G (rs316476530), A137669723C (rs313990885), A137669732G (rs735987765), were selected for marker-trait association analyses (Table 10). Association analysis showed that C137669094T (rs741279417) was significantly (P < 0.05) (P < 0.01) associated with carcass traits and meat quality traits, including small intestine length (SIL) and breast muscle fleshcolor (BMF). The site C137669311G (rs733740424) was significantly (P < 0.05) associated with BMF. The site C137669446T (rs16155707) was significantly associated with chest angle (CA) (P < 0.01) and head width (HW) (P < 0.05). The site A137669484C (rs737463671) was significantly (P < 0.05) associated with CA. The site A137669551G (rs316423136) was significantly (P < 0.05) associated with CW. The site A137669618G (rs731278593) was significantly (P < 0.05) (P < 0.01) associated with SL at various weeks. The site C137669668G (rs317677782) was significantly (P < 0.05) (P < 0.01) associated with leg muscle fleshcolor (LMF) and BMF. Moreover the site A137669723C (rs313990885) and A137669732G (rs735987765) was significantly (P < 0.05) associated with BMF.

**Table 9:** Summary of SNPs identified in the 5' region of chicken *FBXO32* gene.

NO.	SNP^1^	RefSNP	Region	Nucleotide change
1	A137669051T	rs314992955	5'near gene	/
2	C137669076T	rs313186471	5'near gene	/
3	C137669094T	rs741279417	5'near gene	/
4	C137669311G	rs733740424	5'near gene	/
5	C137669446T	rs16155707	5'near gene	/
6	A137669484C	rs737463671	5'near gene	/
7	A137669551G	rs316423136	5'near gene	/
8	A137669618G	rs731278593	5'near gene	/
9	C137669630T	rs315617235	5'near gene	/
10	A137669638T	rs313162537	5'near gene	/
11	C137669668G	rs317677782	5'near gene	/
12	A137669706G	rs316476530	5'near gene	/
13	A137669723C	rs313990885	5'near gene	/
14	A137669732G	rs735987765	5'near gene	/

^1^The number indicated the position on the chromosome 2.

**Table 10:** Association of SNPs in the chicken *FBXO32* gene with the carcass and meat quality traits.

SNP	Traits	P value	Least-square Means ± standard errors (SE)		
C137669094T	SIL(cm)	0.0463	143.61±1.68^a^(TT/123)	141.46±4.27^ab^(CC/11)	135.85±2.41^b^(TC/46)
	BMF	0.0073	56.79±0.96^a^(TT/124)	49.91±3.29^b^(CC/11)	54.70±1.48^ab^(TC/46)
C137669311G	BMF	0.0110	56.44±1.32(CC/81)	55.36±1.26(CG/56)	55.90±1.56(GG/39)
C137669446T	CA(°)	0.0046	60.83±0.58^A^(CC/170)	66.55±3.20^B^(CT/3)	69.45±2.46^B^(TT/8)
	HW(mm)	0.0476	30.79±0.19^a^(CC/163)	27.70±0.94^b^(CT/3)	28.74±0.72^b^(TT/8)
A137669484C	CA(°)	0.0415	61.17±0.60^b^(CC/144)	61.09±1.25^b^(AC/27)	64.73±1.95^a^(AA/7)
A137669551G	CW(mm)	0.0218	66.97±0.83^b^(GG/130)	73.330±1.46^a^(AG/30)	70.97±1.84^a^(AA/19)
A137669618G	SL63(mm)	0.0263	79.28±1.01^b^(GG/47)	87.10±2.09^a^(GA/4)	82.08±2.88^ab^(AA/3)
	SL70 (mm)	0.0433	83.26±0.60^b^(GG/118)	87.05±2.09^a^(GA/13)	82.37±2.33^b^(AA/13)
	SL77 (mm)	0.0070	89.24±1.23^B^(GG/47)	99.03±3.31^A^(GA/4)	92.58±3.36^B^(AA/3)
	SL84 (mm)	0.0041	89.77±1.43^B^(GG/103)	95.52±2.04^A^(GA/12)	89.57±2.61^B^(AA/11)
C137669668G	LMF	0.0140	65.98±0.74^a^(GG/123)	64.33±0.19^a^(GC/43)	58.26±3.01^b^(CC/12)
	BMF	0.0072	57.13±0.87^A^(GG/123)	54.25±1.46^AB^(GC/43)	50.12±2.87^B^ (CC/12)
A137669638T	HW(mm)	0.0314	30.56±0.26^ab^ (TT/64)	30.94±0.22^a^ (TA/59)	30.44±0.27^b^(AA/46)
A137669723C	BMF	0.0128	55.99±1.20^b^(CC/70)	55.15±1.22^b^(AC/59)	59.92±1.49^a^(AA/47)
A137669732G	BMF	0.0147	55.99±1.20^ab^(GG/70)	55.21±1.27^b^ (GA/59)	56.47±1.47^a^(AA/46)

SL63, SL70, SL77 or SL84 indicated shank length at 63, 70, 77 or 84 days, respectively. CA = chest angle. SIL = small intestine length. HW = head width. CW = chest width. BMF = breast muscle fleshcolor. LMF = leg muscle fleshcolor. The ^a,b^ or ^A,B^ values with no common superscripts within a column indicated the comparison was differed significantly (P < 0.05 or P <0.01).

## Discussion

During postnatal growth, skeletal muscle increase occurs mainly due to muscle hypertrophy accompanied by satellite cell proliferation to incorporate new myonuclei into existing myofibers [11]. In this study, the comparison with the highest number of DEGs resulted in the most GO biological process terms, and terms of development process, cell differentiation and cell adhesion were present in multiple comparisons. Lysosome was the most significantly enriched pathway. Lysosomes are a membrane-bound cell organelles that contain acid hydrolase enzymes and are interlinked with endocytosis, phagocytosis and autophagy. Previous research showed that the autophagy-lysosome system was a key player in regulating protein degradation in skeletal muscle [12, 13]. Moreover, other enriched pathways were cell junction-related pathways (focal adhesion, ECM-receptor interaction and cytokine-cytokine receptor interaction), suggesting that pathways critical to maintaining the integrity of tissues might be involved in chicken growth at 7 weeks of age. Focal adhesion complexes serve as mechanical linkages to the ECM and are the signaling centers of numerous intracellular pathways related to cell motility, proliferation and differentiation [14]. ECM components play integral roles in the formation of the muscle satellite cell niche, and their specific interactions with satellite cells can regulate cell adhesion, migration, differentiation, proliferation and self-renewal [15]. Cytokines such as IL-6 are key regulators of cell growth, proliferation, differentiation and apoptosis [16]. Some cytokines produced by myofibers and peritendinous tissue are, termed myokines [17]. Myokines activate of intracellular signaling cascades by binding to their specific receptors, thereby regulating metabolism in skeletal muscle [18].

Importantly, we identified, three crucial transcription factors, CEBPB, FBXO32 and MYOD1, that might be related to chicken growth. Previous research revealed that CEBPB regulated multiple genes in response to GH [19]. Moreover, CEBPB was an activator of adipogenesis and acted as an inhibitor of myogenesis [20, 21]. As one well-known myogenic regulatory factor, MYOD1 has been extensively studied, and its effect on growth has been well-demonstrated [22, 23]. It can trans-differentiate many cell types to muscle cells and is a key regulator of myogenesis [23–25]. Several recently published reports suggested that MYOD1 could modulate and facilitate the assembly of muscle enhancers [26, 27]. Furthermore, CEBPB, FBXO32 and MYOD1 can interact with each other [20, 28]. In this study, network analysis indicated that *FOXO3* was a central gene interacting with *CEBPB*, *FBXO32* and *MYOD1*.

FOXO3 is a member of the Forkhead box class O (FOXO) transcription factor family. Like other members of this family, FOXO3 was demonstrated to play a crucial role in many species, from lower animals to mammals. It performs a variety of cellular functions, including cell growth and differentiation, cell-cycle control, energy metabolism, DNA damage repair, response to oxidative stress, and apoptosis [29–33]. In mammals, *FOXO3* was relatively ubiquitously expressed, consistent with our findings in chicken [34]. However, the mammalian *FOXO3* was predominantly expressed in heart, brain, kidney and ovary, whereas the chicken *FOXO3* was highly expressed in breast muscle and leg muscle tissues [34]. A previous study suggested that FOXO3 contributes to cell growth in striated muscle [33]. Our expression comparison between fast-growing and slow-growing breeds also confirmed its important function in skeletal muscle. It is possible that the higher expression of *CEBPB*, *FBXO32* and *FOXO3* in slow-growing XH chickens contributed to their lower growth performance, whereas the higher level of *MYOD1* would partly counteract these effects. Moreover, a series of genes involved in the somatotropic axis, including *GH*, *GHR*, *IGF1R*, *IGF2R*, *IGF2BP1* and *IGF2BP3*, was effectively up-regulated by FOXO3 knockdown after siRNA interference, suggesting that *FOXO3* might play an important role in growth. In particular, some genes remarkably up-regulated by *FOXO3* knockdown, including *INSR*, *PDK1* and *PDK4*, were present upstream of the IGF1/FOXO signal transduction pathway, which would further promote FOXO3 inhibition [33, 34]. Recent studies suggested that MYOD was a direct target of FOXO3 in myoblasts [35]. In vivo and in vitro, *FOXO3* was demonstrated to play an important role in activating *MYOD* transcription [35]. In this study, *FOXO3* knockdown resulted in a significant down-regulation of *MYOD*, whereas expression of the other MRFs (*MYF5* and *MYF6*) was unaffected, consistent with previously published studies [35]. These results indicated that *FOXO3* could interact with *MYOD* but not the others MRFs. Conversely, previous research identified FOXO3 as a major activator of *FBXO32* expression, a factor associated with skeletal muscle protein degradation [36, 37]. In contrary, we found that *FOXO3* knockdown strongly increased *FBXO32* expression. Nevertheless, our data revealed that *FOXO3* down-regulation induced a dramatic down-regulation of *CEBPB*, which is an inhibitor of myogenesis [20]. The up/down-regulation of *FBXO32* and *CEBPB* was inconsistent with the reported inhibitory roles of FOXO3 on muscle growth. Therefore, further study is needed to investigate protein expression changes. The human FOXO3 contains a sequence of 95 amino acids forming a forkhead DNA-binding domain motif (148–257 amino acids). It also possesses a nuclear localization sequence (NLS, 249–251 and 269–271 amino acids), a nuclear export sequence (NES, 386–396 amino acids) and a conserved C-terminal transactivation domain [38]. The sequences of the chicken *FOXO3* gene showed high identity with the previously cloned mammalian FOXO3. The chicken FOXO3 had the same amino acids as the human FOXO3 in the NLS and NES. It shared high homology with human FOXO3 in the forkhead DNA-binding domain, with a six amino acid difference.

FBXO32, also known as Atrogin 1 or MAFbx, is a skeletal and cardiac muscle-specific F-box containing protein that was shown to be associated with the maintenance of muscle mass [22, 25]. *FBXO32* is a muscle-specific gene that has a key function in muscle atrophy [39]. Previous research on feed deprivation showed that as the muscle degraded, *FBXO32* expression increased [40]. Although we did not find any association between SNPs of *FBXO32* and chicken growth traits, our findings showed that it was highly expressed in chicken leg muscle, heart and breast muscle tissues, which is in accordance with its role. *FBXO32* was regulated by multiple transcription factors. A previous study suggested that FOXO3 can act on the promoter of *FBXO32*, mediating *FBXO32* transcription and muscle cell atrophy [41]. After *FBXO32* expression significantly decreased, growth related genes including *PDK4*, *IGF2R* and *IGFBP3* were significantly down-regulated. Previous published reports showed that FBXO32 and PDK4 may interact in skeletal muscle metabolism [42, 43]. The expression levels of *GH* and *GHR* had tendency to increase, indicating that *FBXO32* may have influence on muscle growth at the mRNA level.

Thus far, many SNPs and QTLs were reported to correlate with growth [3, 4]. In humans, one *FOXO3* promoter SNP was associated with human body mass index [44]. In pig, *FOXO3* SNPs were closely associated with growth and development traits [45]. To date, this study is the first to scan variations in the chicken *FOXO3* gene and then evaluate their effects on chicken growth. In humans, FOXO3 was phosphorylated and acetylated on multiple sites, including K242, K245, K259, K271, K290, K569, S207, S253, S295, S315, S318, S321, S325, S345, S399, S413, S426, S555, S588, S626, S644, T32 and T179 [33, 38, 46]. In this study, we scanned the entire exon 2 of chicken *FOXO3*, and a total of 11 SNPs were identified in the 1,432 bp exon 2 fragment. These variation sites corresponded to R222, P238, A251, S286, N316, D339, P361, T404, F459, T475 and N530 in human FOXO3. Although these variations were not present in phosphorylation or acetylation sites, C66716002T (corresponding to A251 in human FOXO3), was located in the forkhead DNA-binding domain, which is crucial in regulating the transcription of target genes. This SNP did not cause an amino acid change, but might affect mRNA splicing, stability, or protein folding, and thereby alter protein functions such as DNA-binding [47]. Moreover, it is located in the NLS region. Our association analysis demonstrated that this mutation had a significant effect on growth traits. Among the mutations identified in exon 2 of chicken *FOXO3*, G66715314C was a non-synonymous variation resulting in a change in the amino acid sequence. It was previously shown that non-synonymous SNPs affect protein functions and protein-protein interactions [48, 49]. Therefore, the G66715314C mutation may have important effects on economic traits, and its association with chicken growth traits needs further study. Conversely, four SNPs found in this study were located near the exon-intron boundaries, which are special regions that usually had important functional roles in protein [50]. Three of these four SNPs were associated with growth traits. Importantly, the G66716193A site, which was 60 bp away from the intron-exon boundary, was significantly associated with most growth traits.

In summary, this study provided a comprehensive transcriptome analysis of breast muscle between fast- and slow-growing chickens. *CEBPB*, *FBXO32*, *FOXO3* and *MYOD1* may play key roles in chicken growth at seven weeks of age. Further expression analysis, siRNA analysis and association analysis demonstrated that chicken *FOXO3* is a candidate gene involvement in chicken growth. Our observations provide new clues to understand the molecular basis of chicken growth.

## Materials and Methods

### Ethics statement

All animal experiments were performed in accordance with and were approved by the Animal Care Committee of South China Agricultural University (Guangzhou, China) (approval ID: SCAU#0011). All efforts were made to minimize animal suffering.

### Animals and tissues

Two chicken breeds WRR and XH were used for RNA-Seq in this study. WRR is a breed with a fast growth rate, while XH is a Chinese native breed with a slow growth rate. WRR and XH were provided by Guangdong Wens Foodstuff Company Ltd, Guangdong, China and Zhicheng Avian Breeding Company Ltd, Guangdong, China, respectively. Briefly, birds were fed *ad libitum* with 16.5% CP and 2800 kcal of ME/kg, with free access to water. BW was measured at seven weeks of age and three female chickens from each of the two-tail samples of WRR and XH were selected based on it. The four groups WRR with high weight (WRR_h_), WRR with low weight (WRR_l_), XH with high weight (XH_h_) and XH with low weight (XH_l_) were generated. Their average BW7 values were 1064.0 ± 11.1, 695.0 ± 24.4, 305.8 ± 23.3 and 207.6 ± 11.1 g, respectively. At 7 weeks of age, chickens were killed by stunning followed by exsanguination. Breast muscles were collected and stored at -80°C until RNA extraction.

Five XH chickens at 7 weeks of age were used for expression analysis of *FOXO3* and *FBXO32* in different tissues. A total of 19 tissues, cerebrum, cerebellum, pituitary, hypothalamus, heart, liver, spleen, lung, kidney, breast muscle, leg muscle, muscular stomach, glandular stomach, ovary, duodenum, subcutaneous fat, abdominal fat, thymus and bursa of Fabricius, were collected from each chicken.

Four XH and four WRR chickens with normal BW were used for the expression analysis between different breeds by qPCR. Breast muscle and leg muscle tissues were dissected from those 8 birds. All those birds were obtained from the Chicken Breeding Farm of South China Agricultural University, Guangdong, China.

An F2 full sibling hybrid population, from a WRR and XH cross as described previously, was used for association analysis between SNPs and chicken growth or carcass traits [51]. Genomic DNA was extracted from EDTA-anticoagulated blood and then used for genotyping.

### RNA extraction and RNA-Seq

Total RNA from various tissues and DF-1 cells was isolated by TRIzol reagent (Invitrogen, Carlsbad, CA, USA) and then treated with DNase (Promega, Madison, WI, USA) following the manufacturer’s instructions. RNA quality and concentration were evaluated by an Agilent 2100 Bioanalyzer (Agilent Technologies, Santa Clara, CA, USA). Subsequently, three breast muscle RNAs of the same group were mixed in equal amounts. In this way, four pooled samples (WRR_h_, WRR_l_, XH_h_ and XH_l_) were generated and then were sent to Shanghai Majorbio Bio-pharm Biotechnology Co., Ltd. (Shanghai, China) for RNA-Seq. cDNA libraries were constructed according to Illumina's protocols and then each library was sequenced on a single line of Illumina Hiseq 2000 (Illumina, San Diego, CA, USA) to obtain paired-end 101-bp reads.

### Bioinformatic analysis of RNA-Seq

For raw data from RNA-Seq, we first removed reads containing adaptors, unknown bases and low quality bases to obtain high quality reads. Then, the clean reads of the four samples were aligned to the chicken reference genome (http://asia.ensembl.org/info/data/ftp/index.html) by TopHat software, with no more than 2 bp mismatches. The mapped reads were used for further transcriptome annotation and expression calculation with using FPKM (Fragments Per Kilobase of transcript per Million mapped reads). All expressed genes were subjected to GO analysis by AmiGO2 (http://amigo.geneontology.org/amigo), with Bonferroni-adjusted P value < 0.05. With the use of Cuffdiff (http://cufflinks.cbcb.umd.edu/), genes with greater than 2-fold changes between two samples and a q value < 0.05 were regarded as DEG. All those DEGs were subjected to GO analysis and KEGG pathway enrichment analysis with the DAVID Functional Annotation Tool (http://david.abcc.ncifcrf.gov/) using a 0.05 cutoff for the P value and Benjiamini adjusted P < 0.1 cutoff for the Benjamini adjusted p-value. Moreover, all DEGs underwent gene network analysis by IPA.

### cDNA synthesis and qPCR

Primers P1 to P5 (S6 Table) were used for the validation of RNA-Seq data by qPCR. For the *FOXO3* expression analysis in different tissues and breeds, total RNA was reverse transcribed using a PrimeScript RT reagent Kit with gDNA Eraser (Takara Co., Japan) to synthesize the first-strand cDNAs. qPCR was conducted in a total volume of 20 μL: 10 μL Bestar Real time PCR Master Mix (SYBR Green) (DBI Bioscience, Germany), 0.5 μL of each primer (10 μM), 8.0 μL of RNase-free water and 1 μL of cDNA on a BIO-RAD CFX96 system (Bio-Rad, USA). P4 was used for qPCR of *FOXO3* and β-actin (P1) was used as the internal control (S6 Table). All reactions were run in triplicates. The relative expression level was calculated by the 2^-ΔΔCt^ method. Where ΔΔCt corresponded to the difference between ΔCt measured for the mRNA level of each sample and ΔCt measured for the mRNA level of the reference sample, ΔCt = Ct_target gene_−Ct_reference gene_.

### Cloning of the chicken *FOXO3* gene

Total RNA was extracted from the breast muscle of an XH chicken. According to the predicted partial cDNA sequences of the chicken *FOXO3* gene (NCBI accession number: XM_001234495.3), a pair of primers (P6) was designed and primer-directed RT-PCR was used to amplify a partial cDNA sequence and part of the 3’UTR region (S6 Table). The products were subcloned into the pMD18-T vector (TaKaRa Biotechnology Co Ltd, Dalian, China) and then sequenced on Applied Biosystem model 3730 sequencer. Based on the obtained sequences, P7 and P8 (S6 Table) were designed for 3’ RACE. With the use of the SMART RACE cDNA amplification kit (Clontech Laboratories, Mountain View, CA), a breast muscle cDNA library was prepared and the 3’ ends of the cDNA were amplified by specific forward primers and random reverse primers. Sequence analysis was conducted using DNASTAR software (http://www.dnastar.com). The homology of FOXO3 among different species, including human (NP_001446.1), mouse (NP_062714.1), rat (NP_001099865.1), elephant (XP_003404353.1), whale (XP_004264796.1), pig (NP_001129431.1), dog (XP_003639448.1), sheep (NP_001254818.1), cattle (NP_001193012.1), chicken, Xenopus (NP_001086418.1) and fish (XP_003449931.1), was analyzed with MEGA 6 software (http://www.megasoftware.net/).

### Polymorphism identification in *FOXO3* and *FBXO32* and association analysis with growth traits

With the use of P9, P10 and P11 (S6 Table), ten chickens from the WRR and XH F2 full sibling hybrid population were selected to identify polymorphisms in the coding region and intron region near the exon-intron boundaries of the *FOXO3* gene through direct sequencing. Primers P30 (S6 Table) were used for identify polymorphisms in the 5' flank region of the *FBXO32* gene. Another ten chickens from the WRR & XH F2 full sib hybrid population were selected to sequence. Sequence analyses were conducted with the software DNASTAR V 3.0 (http://www.biologysoft.com/; Steve ShearDown, 1998–2001 version reserved by DNASTAR Inc., Madison, Wisconsin, USA). Only polymorphisms occurring more than twice were regarded as variations. P10 and P30 was also used for the following association analyses in this population by direct sequencing. Association analyses of polymorphisms with chicken growth and carcass traits were conducted using SAS 8.0 software (SAS Institute Inc, Cary, NC, USA) using the following GLM model:

Y=μ+G+D+H+S+e

where Y is an observation of the traits, μ is the overall population mean, G is the fixed effect of genotype, D is the random effect of dam, H is the fixed effect of hatch, S is the fixed effect of sex, and e is the residual error.

### siRNA design, cell culture and transfection

siRNA molecules were synthesized by Shanghai GenePharma Co. Ltd (Shanghai, China) with commercial service. Three FOXO3 target sites were selected: siRNA-703 (sense: 5’gccggauggaagaauucaatt3’, antisense: 5’ uugaauucuuccauccggctt3’), siRNA-842 (sense: 5’gcgccguguccauggacaatt3’, antisense: 5’uuguccauggacacggcgctt3’) and siRNA-1082 (sense: 5’gcaccgagcuggaugaugutt3’, antisense: 5’acaucauccagcucggugctt3’). A NC siRNA fragment was produced as the control. Three specific siRNAs targeting FBXO32 were synthesized.: siRNA-001 (sense: 5’ ccuucaacagacuugacuu 3’, antisense: 5’ ggaaguugucugaacugaa 3’), siRNA-002 (sense: 5’ gcaacugaacaacauucaa 3’, antisense: 5’ cguugacuuguuguaaguu3’), siRNA-003 (sense: 5’ ggcuaauccuaucugacaa 3’, antisense: 5’ ccgauuaggauagacuguu 3’).

The chicken fibroblast cell line DF-1 was obtained from the Cell Bank of Committee on Type Culture Collection of Chinese Academy of Sciences and was cultured in Dulbecco’s Modified Eagles Medium (DMEM) (GIBCO BRL, Life Technologies, Invitrogen Corporation, Carlsbad, CA) supplemented with 10% fetal bovine serum (FBS) (Hyclone Laboratories, Logan, Utah), 100 U/ml penicillin, and 100 μg/ml streptomycin. Cells were cultured and maintained in humidified air at 37°C with 5% CO_2_. When the DF-1 cells were grown to approximately 50–80% confluence, independent transfections of the three siRNAs and the control NC siRNA were carried out using the transfection reagent Lipofectamine 3000 (Invitrogen Corporation, Carlsbad, CA) following the manufacturer’s protocol. For each siRNA targeting *FOXO3*, a plasmid concentration gradient (25 nM, 50 nM and 100 nM) was set to investigate which concentration ratio had the highest transfection efficiency. Here, experiments for each gradient were conducted in triplicates. For each siRNA targeting *FBXO32*, the recommended concentration of Lipofectamine 2000 (50 nM) was transfected into DF-1 cells. At 48 h post-transfection, RNA was extracted from cells to perform qPCR. For each siRNA, the expression of target genes at each concentration was compared to that in cells transfected with NC siRNA. P12 was used for qPCR for *FOXO3*, P14 was used for qPCR for *FBXO32*, and *β-actin* (P1) was used as the control (S6 Table). Eventually, we found that siRNA-1082 at 100 nM had the highest relative efficiency of transfection and it was selected for the following experiments. siRNA-002 for *FBXO32* had the highest relative efficiency of transfection and was used for transfecting DF-1 cells. When cells were grown to 50–80% confluence, siRNA-1082 and NC siRNA at 100 nM and siRNA-002 and NC siRNA at 50 nM were transfected into DF-1 cells. RNA was collected at 48 h post-transfection. With the use of qPCR, the expression change was analyzed for growth-related genes including *CEBPB*, *FBXO32*, *GH*, *GHR*, *IGF1R*, *IGF2R*, *IGFBP2*, *IGFBP3*, *IGF2BP1*, *IGF2BP3*, *INSR*, *MYF5*, *MYF6*, *MYOD*, *MYH10*, *PDK1* and *PDK4* (primers are shown in S6 Table).

## Supporting Information

S1 Fig Sequence length distribution of genes identified in WRR_h_, WRR_l_, XH_h_ and XH_l_. (DOC)

S2 Fig Directionality of DEGS. WRR_h_ vs. WRR_l_, XH_h_ vs. XH_l_, WRR_h_ vs. XH_h_ and WRR_l_ vs. XH_l_ indicate the comparisons between WRR_h_ and WRR_l_, between XH_h_ and XH_l_, between WRR_h_ and XH_h_ and between WRR_l_ and XH_l_, respectively. In each comparisons, up-regulated indicates that the expression in the second group was higher than that in the first group, while down-regulated indicates that the expression in the first group was higher than that in the second group.
(DOC)

S3 Fig The cDNA sequences of the two transcripts of the chicken *FOXO3* gene. Sequences in the black square frame indicated the sequences absent in the second transcript. Sequences in the CDS are presented in uppercase, while those in the 3’ UTR are presented in lowercase.
(DOC)

S4 Fig Cell proliferation pre-transfection, 24 h post-transfection and 48 h post-transfection. A: DF-1 cells. B: Skeletal muscle cells.
(TIF)

S1 Table Detailed information on DEGs in the four comparisons of WRR_h_ vs. WRR_l_, XH_h_ vs. XH_l_, WRR_h_ vs. XH_h_ and WRR_l_ vs. XH_l._
(XLS)

S2 Table DEGs overlapped in comparisons of WRR_h_ vs. WRR_l_ and XH_h_ vs. XH_l._
(XLS)

S3 Table DEGs overlapped in comparisons of WRR_h_ vs. XH_h_ and WRR_l_ vs. XH_l._
(XLS)

S4 Table DEGs that were uniquely expressed in one of the two samples in each comparisons. (DOC)

S5 Table The GO BP ALL category for DEGs in each comparisons of WRR_h_ vs. WRR_l_, XH_h_ vs. XH_l_, WRR_h_ vs. XH_h_ and WRR_l_ vs. XH_l._
(XLS)

S6 Table Descriptions of all primers used in this study. (DOC)
