# Supplementary figures and images for: A Genome-Wide mRNA Screen and Functional Analysis Reveal FOXO3 as a Candidate Gene for Chicken Growth
Source: PLoS One. 2015 Sep 14;10(9):e0137087. doi: 10.1371/journal.pone.0137087 (PMC4569328; doi:10.1371/journal.pone.0137087)

**Fig S1. Sequence length distribution of genes identified in WRRh, WRRl, XHh and XHl.**


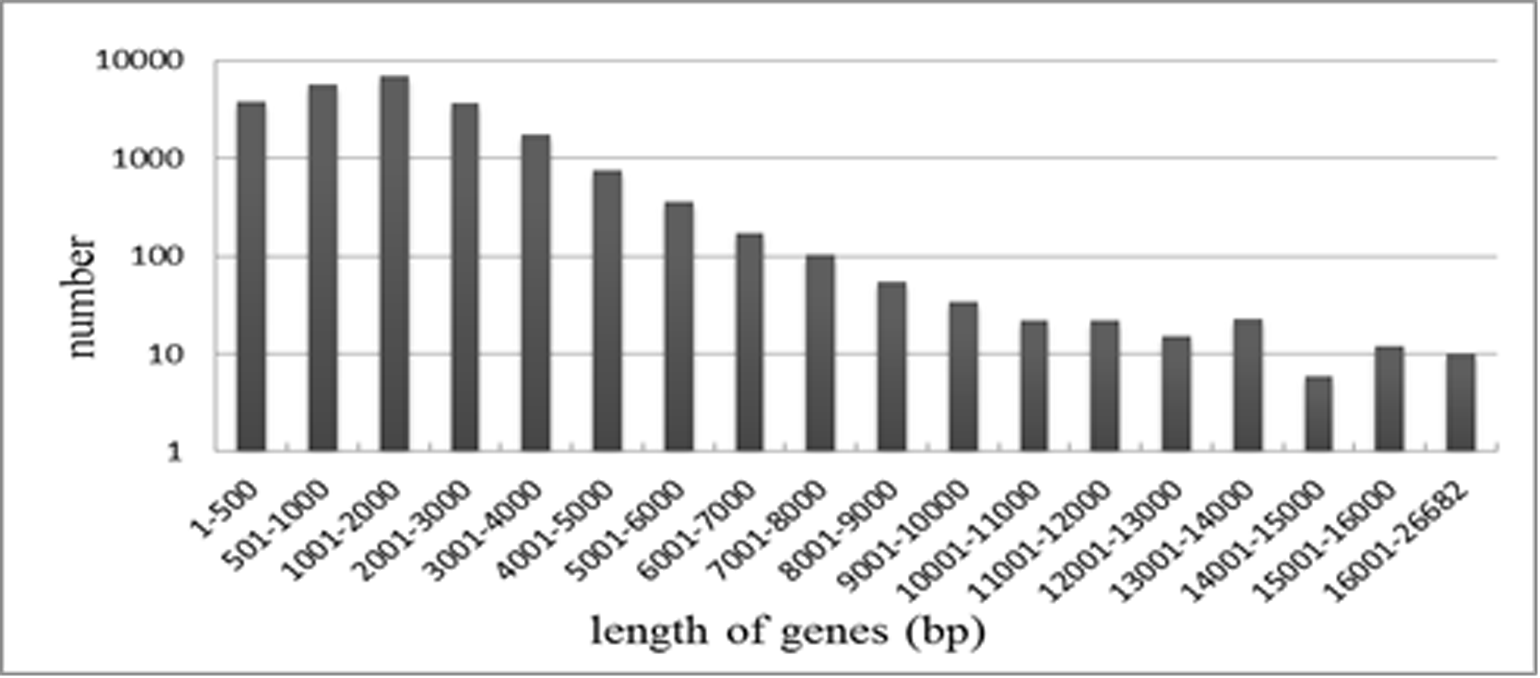

Supplement: S1 Fig — (DOC) [file pone.0137087.s001.doc]

**Fig S2. Directionality of DEGS**.


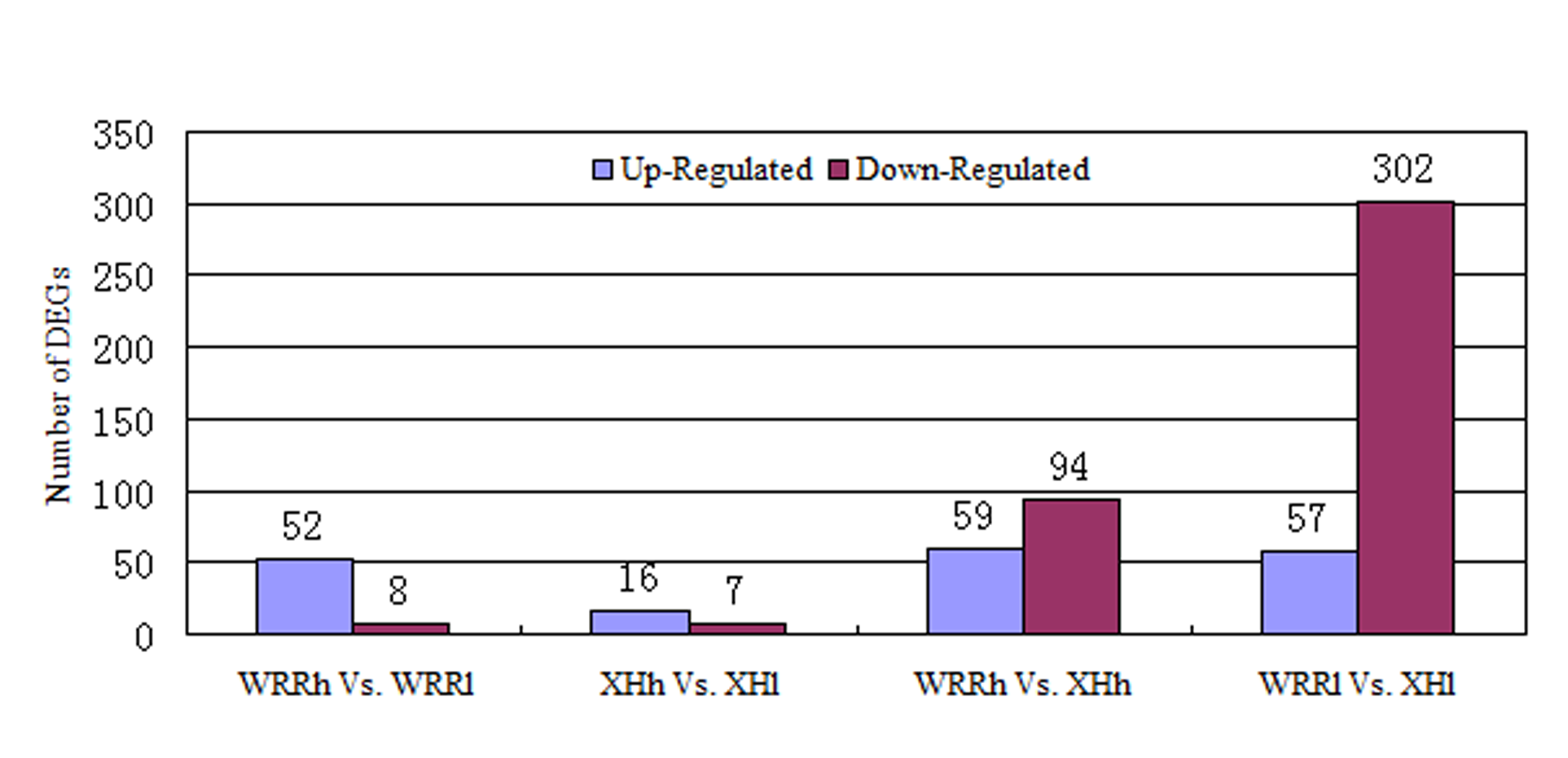

Supplement: S2 Fig — WRRh vs. WRRl, XHh vs. XHl, WRRh vs. XHh and WRRl vs. XHl indicate the comparisons between WRRh and WRRl, between XHh and XHl, between WRRh and XHh and between WRRl and XHl, respectively. In each comparisons, up-regulated indicates that the expression in the second group was higher than that in the first group, while down-regulated indicates that the expression in the first group was higher than that in the second group. (DOC) [file pone.0137087.s002.doc]

**Fig S3. The cDNA sequences of the two transcripts of the chicken *FOXO3* gene.**


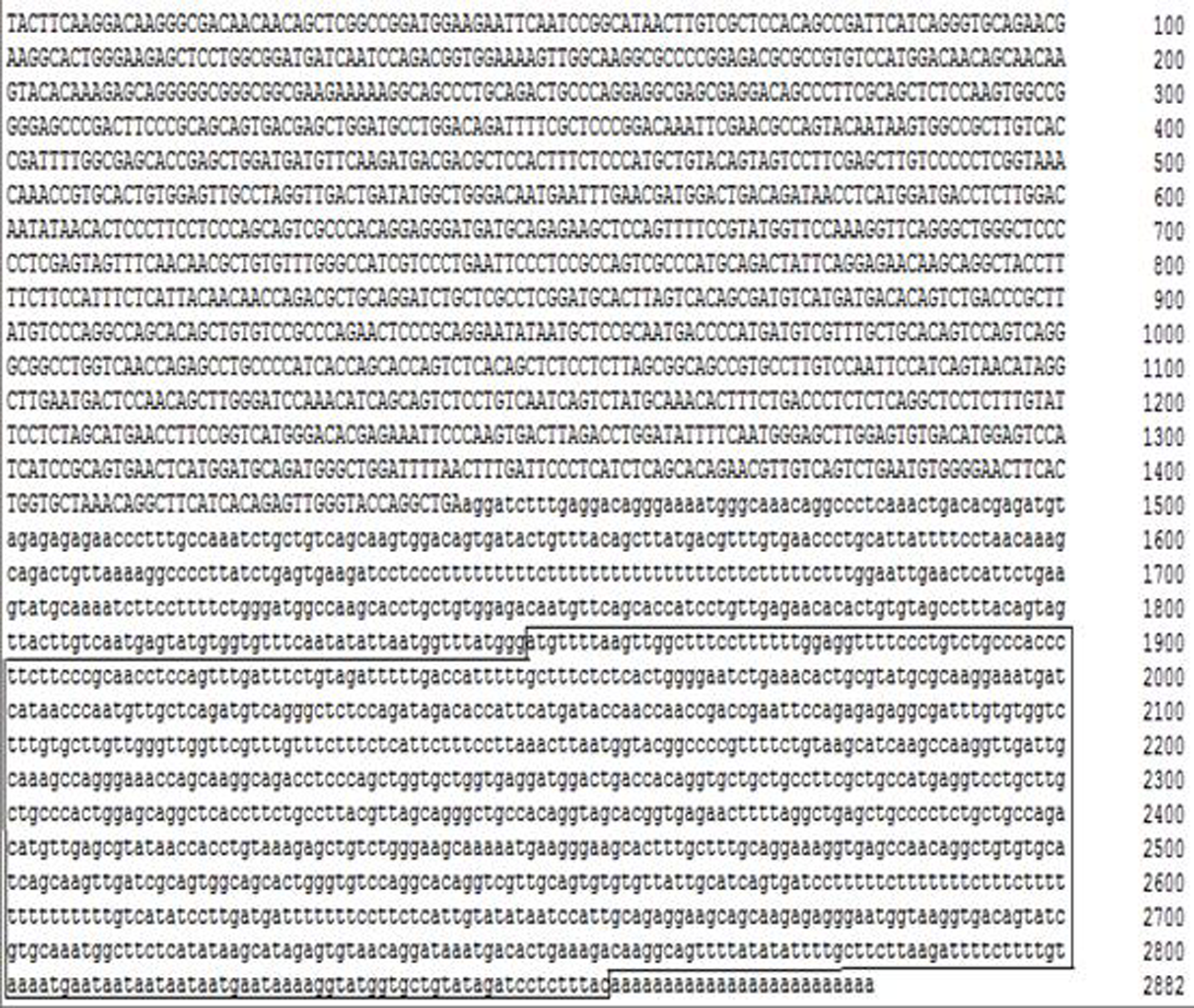

Supplement: S3 Fig — Sequences in the black square frame indicated the sequences absent in the second transcript. Sequences in the CDS are presented in uppercase, while those in the 3’ UTR are presented in lowercase. (DOC) [file pone.0137087.s003.doc]

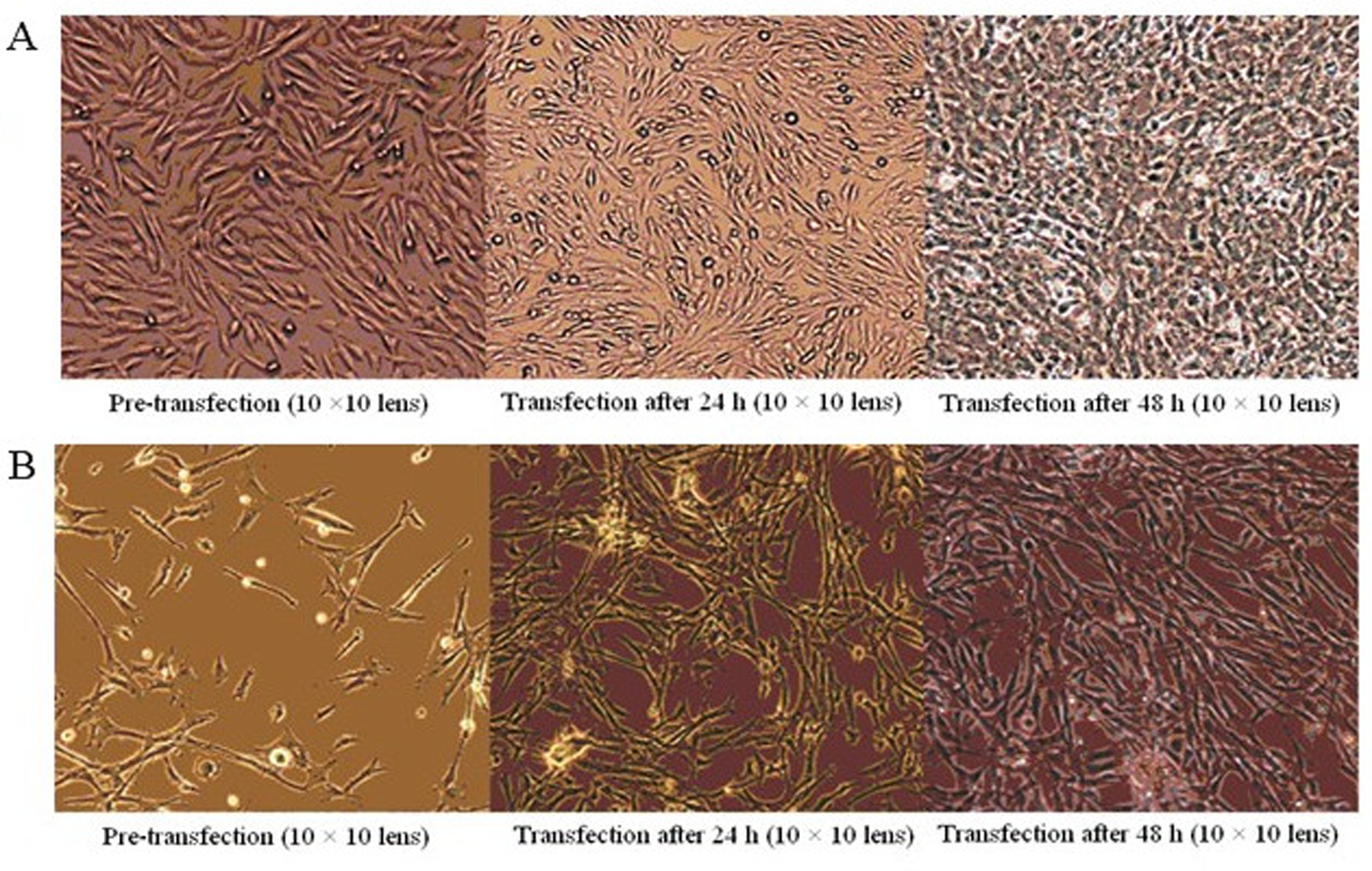

Supplement: S4 Fig — A: DF-1 cells. B: Skeletal muscle cells. (TIF) [file pone.0137087.s004.tif]
