# Supplementary material for: A Genome-Wide mRNA Screen and Functional Analysis Reveal FOXO3 as a Candidate Gene for Chicken Growth
Source: PLoS One. 2015 Sep 14;10(9):e0137087. doi: 10.1371/journal.pone.0137087 (PMC4569328; doi:10.1371/journal.pone.0137087)
Supplement: S4 Table — (DOC) [file pone.0137087.s008.doc]

**Table S4. DEGs that were uniquely expressed in one of the two samples in each comparisons.**

| Contrasts |  | Gene name | Description |
| --- | --- | --- | --- |
| WRR_h_Vs.WRR_l_ | WRR_h_ | *DAGLB* | diacylglycerol lipase, beta |
|  |  | *HEATR3* | HEAT repeat containing 3 |
|  |  | *C9orf41* | chromosome Z open reading frame |
|  | WRR_l_ | *SCRIB* | Scribbled planar cell polarity protein |
|  |  | *EIF3J* | eukaryotic translation initiation factor 3, subunit J |
|  |  | *GSPT1* | G1 to S phase transition 1 |
| XH_h_ Vs. XH_l_ | XH_h_ | *ABCG2* | ATP-binding cassette, sub-family G (WHITE), member 2 |
|  |  | *C4H4orf41* | chromosome 4 open reading frame, human C4orf41 |
|  |  | *FOXP2* | forkhead box P2 |
|  |  | *PAM16* | coronin 7 |
|  | XH_l_ | *ATP11B* | ATPase, class VI, type 11B |
|  |  | *C11orf2* | Vacuolar protein sorting-associated protein 51 homolog |
|  |  | *NUP107* | Nucleoporin 107kDa |
|  |  | *PLEKHG1* | Pleckstrin homology domain containing, family G (with RhoGef domain) member 1 |
|  |  | *TRIM24* | Tripartite motif containing 24 |
|  |  | *TTR* | transthyretin |
| WRR_h_ Vs. XH_h_ | WRR_h_ | *LOC429682* | GTPase IMAP family member 7-like |
|  |  | *ANGPT2* | Angiopoietin-2 |
|  |  | *ATP11B* | ATPase, class VI, type 11B |
|  |  | *GIGYF2* | GRB10 interacting GYF protein 2 |
|  |  | *MMP7* | matrix metallopeptidase 7 (matrilysin, uterine) |
|  |  | *TTR* | transthyretin |
|  | XH_h_ | *SCRIB* | Scribbled planar cell polarity protein |
|  |  | *C4H4orf41* | chromosome 4 open reading frame, human C4orf41 |
|  |  | *FOXP2* | forkhead box P2 |
|  |  | *GSPT1* | G1 to S phase transition 1 |
|  |  | *SGMS1* | sphingomyelin synthase 1 |
| WRR_l_ Vs. XH_l_ | WRR_l_ | *EX-FABP* | lipocalin 8 |
|  |  | *ABCG2* | ATP-binding cassette, sub-family G (WHITE), member 2 |
|  |  | *ANGPT2* | Angiopoietin-2 |
|  |  | *ATP13A2* | ATPase type 13A2 |
|  |  | *FAF1* | Fas (TNFRSF6) associated factor 1 |
|  | XH_l_ | *C11orf2* | Vacuolar protein sorting-associated protein 51 homolog |
|  |  | *DAGLB* | diacylglycerol lipase, beta |
|  |  | *HEATR3* | HEAT repeat containing 3 |
|  |  | *PLEKHG1* | Pleckstrin homology domain containing, family G (with RhoGef domain) member 1 |
|  |  | *C9orf41* | chromosome Z open reading frame |
|  |  | *TRIM24* | Tripartite motif containing 24 |
|  |  | *TSC22D1* | TSC22 domain family, member 1 |
