# Supplementary material for: A Genome-Wide mRNA Screen and Functional Analysis Reveal FOXO3 as a Candidate Gene for Chicken Growth
Source: PLoS One. 2015 Sep 14;10(9):e0137087. doi: 10.1371/journal.pone.0137087 (PMC4569328; doi:10.1371/journal.pone.0137087)
Supplement: S6 Table — (DOC) [file pone.0137087.s010.doc]

**Table S6. Descriptions of all primers used in this study.**

| Gene | Primer | Sequence(5’ to 3’) | Annealing temp (◦C) | Product size (bp) | Purpose |
| --- | --- | --- | --- | --- | --- |
| β-actin | P1 | F:ccccatgccatcctccgtctg | 56/63 | 223 | Validation of RNA-seq by qPCR |
|  |  | R:cctcggggcacctgaacctctc |  |  |  |
| RLP29 | P2 | F:ggcgctgtctgctgtgggtt | 56 | 191 |  |
|  |  | R:agccccttcttgttgtgtttcttg |  |  |  |
| PDK4 | P3 | F:cccggatgctgatgaaccaacaca | 63 | 179 |  |
|  |  | R:aagcttcagttctggagacgttaa |  |  |  |
| FOXO3 | P4 | F:tacttcaaggacaagggcgac | 63 | 219 |  |
|  |  | R:ccccctgctctttgtgtact |  |  |  |
| LAPTM5 | P5 | F:accaaacccccaaaatgctg | 56 | 239 |  |
|  |  | R:ttcaccaccccaaagaggag |  |  |  |
| FOXO3 | P6 | F:tacttcaaggacaagggcgac | 56 | 1472 | cDNA cloning |
|  |  | R:tgcccattttccctgtcctc |  |  |  |
| FOXO3 | P7 | tgggagcttggagtgtgacatg | 55 | / | 3’RACE |
|  | P8 | tgggctggattttaactttgat | 58 | / |  |
| FOXO3 | P9 | F:atgggtggtgaatgtgtgtgac | 58 | 1125 | SNP identification |
|  |  | R:gggcgggagaaaggaaataa |  |  |  |
| FOXO3 | P10 | F:ccagccttcccatttccatca | 58 | 1057 |  |
|  |  | R:cgggagttctgggcggacac |  |  |  |
| FOXO3 | P11 | F:cgcctcggatgcacttagtca | 58 | 627 |  |
|  |  | R:tgcccattttccctgtcctc |  |  |  |
| FOXO3 | P12 | F: cccgacttcccgcagcag | 62 | 187 | siRNA |
|  |  | R: gggggacaagctcgaaggact |  |  |  |
| CEBPB | P13 | F: tggaagtggctaatttctattacga | 58.5 | 141 |  |
|  |  | R: ccagataggggctgaagtcaatg |  |  |  |
| FBXO32 | P14 | F: ggcgacctcagcagcttttgtaa | 58 | 179 |  |
|  |  | R: cgctcttttgtgctccccttgt |  |  |  |
| GH | P15 | F: tgccgagacatataaagagttc | 56 | 107 |  |
|  |  | R: gagctgggatggtttctgagta |  |  |  |
| GHR | P16 | F:cctgatccccctgtgcacctta | 63 | 203 |  |
|  |  | R:ggaaccactgttgagagcctgg |  |  |  |
| IGF1R | P17 | F: ttcaggaaccaaagggcga | 63 | 158 |  |
|  |  | R: tgtaatctggagggcgatacc |  |  |  |
| IGF2R | P18 | F: cgtgggaagcaattgatacaga | 63 | 203 |  |
|  |  | R: gccttgctgggaacatttactt |  |  |  |
| IGFBP2 | P19 | F: ctgccggatgagcgaggtc | 60 | 166 |  |
|  |  | R: gcgcaccctggatcacctt |  |  |  |
| IGFBP3 | P20 | F: cacggcgggagacaagctg | 60 | 192 |  |
|  |  | R: ttggcttgctctttcctgatgat |  |  |  |
| IGF2BP1 | P21 | F: catggaaaacagcttgagatt | 60 | 148 |  |
|  |  | R: cttgctcacagttttctacagt |  |  |  |
| IGF2BP3 | P22 | F: ggcaataaggaccaggctagac | 63 | 156 |  |
|  |  | R: gcccctctgtccaaatcca |  |  |  |
| INSR | P23 | F: agccctgaagacaaacggtgac | 60 | 125 |  |
|  |  | R: cacggaaatcaggaggccaata |  |  |  |
| MYF5 | P24 | F: tgaggaacgccatcaggt | 58 | 141 |  |
|  |  | R: gcgagtccgccatcacat |  |  |  |
| MYF6 | P25 | F: cgccatcagctacatcgagag | 58 | 144 |  |
|  |  | R: ccgcaggtgctcaggaagt |  |  |  |
| MYOD | P26 | F: atcaccaaatgacccaaagc | 58 | 149 |  |
|  |  | R: gggaacagggactcccttca |  |  |  |
| MYH10 | P27 | F:ggtggaagacatggcagaattaac | 63 | 185 |  |
|  |  | R: ggcgcttcttccctctgtacat |  |  |  |
| PDK1 | P28 | F: tgtgccgtcccacctctatc | 63 | 164 |  |
|  |  | R: cacctccgccacgatcactc |  |  |  |
| PDK4 | P29 | F:cccggatgctgatgaaccaacaca | 63 | 179 |  |
|  |  | R:aagcttcagttctggagacgttaa |  |  |  |
| FOXO3 | P30 | F:gtgggaaaaagcataaac | 50 | 794 | SNP identification |
|  |  | R: atctgcctgcctgaactc |  |  |  |
